# Identification of N6-methyladenosine-associated ferroptosis biomarkers in cervical cancer

**DOI:** 10.1186/s41065-025-00418-3

**Published:** 2025-04-07

**Authors:** Jinzhe Liu, Buwei Han, Xijiao Hu, Mengke Yuan, Zhiwei Liu

**Affiliations:** 1 Department of Chinese Pharmacy, School of Pharmacy, Guizhou University of Traditional Chinese Medicine, Guiyang, Guizhou China; 2 Department of Chinese Pharmacy, School of Pharmacy, Harbin University of Commerce, Harbin, Heilongjiang China; 3 Postdoctoral Scientific Research Workstation, Harbin University of Commerce, Harbin, Heilongjiang China; 4 Department of Obstetrics and Gynecology, The Second Affiliated Hospital of Heilongjiang University of Chinese Medicine, Harbin, Heilongjiang China; 5 Department of Gynaecology and Obstetrics, The First Affiliated Hospital of Zhejiang Chinese Medical University (Zhejiang Provincial Hospital of Chinese Medicine), Hangzhou, Zhejiang China; 6 Department of Pediatrics, The Second Affiliated Hospital of Guizhou University of Traditional Chinese Medicine, No. 83, Feishan Road, Guiyang, Guizhou Province China

**Keywords:** Cervical cancer, m6A, Ferroptosis, Biomarker, Nomogram

## Abstract

**Background:**

Cervical cancer (CC) stands as a major contributor to female mortality. The pathogenesis of CC is linked with various factors. Our research aimed to unravel the underlying mechanisms of ferroptosis and m6A RNA methylation in CC through bioinformatics analysis.

**Methods:**

Three CC datasets, including GSE9750, GSE63514, and TCGA-CESC, were incorporated. m6A-related genes were derived from published sources, while ferroptosis-related genes were obtained from the FerrDb database. Differential expression and correlation analyses were performed to identify differentially expressed m6A-related ferroptosis genes (DE-MRFGs) in CC. Subsequently, the biomarkers were further identified using machine learning techniques. Gene Set Enrichment Analysis (GSEA) and Kaplan–Meier (KM) survival analysis were also performed to comprehend these biomarkers. Furthermore, a competing endogenous RNAs (ceRNA) network involving biomarkers was established. Finally, biomarkers expression were verified by real-time quantitative polymerase chain reaction (RT-qPCR).

**Results:**

From the DE-MRFGs, six genes, including *ALOX12*,* EZH2*,* CA9*,* CDCA3*,* CDC25A*,* HSPB1*, were selected. A nomogram constructed based on these biomarkers exhibited potential clinical diagnostic value for CC, with good diagnostic accuracy confirmed through calibration curves. GSEA unveiled associations of these biomarkers with cell proliferation, spliceosome, and base excision repair. KM survival analysis demonstrated significant differences in survival outcomes between high and low expressions of *HSPB1*,* EZH2*, and *CA9* samples. A ceRNA network was constructed involving three biomarkers, such as *CDC25A*,* CDCA3*, and *EZH2*, 29 miRNAs, and 25 lncRNAs. In RT-qPCR verification, the expression of *ALOX12*, *EZH2* and *CDC25A* was significantly higher in CC samples, while *HSPB1* expression was higher in control samples.

**Conclusion:**

Six genes, namely *ALOX12*,* EZH2*,* CA9*,* CDCA3*,* CDC25A*, and *HSPB1*, were identified as m6A-regulated ferroptosis biomarkers in CC. These findings offer valuable insights into disease pathogenesis and hold promise for advancing CC treatment and prognosis.

**Supplementary Information:**

The online version contains supplementary material available at 10.1186/s41065-025-00418-3.

## Introduction

Cervical cancer (CC) ranks as the fourth leading cause of female mortality globally [1]. Despite the decrease in CC morbidity due to formalized screening programs and widely available vaccinations, the assessment of prognosis and improvements in survival rates for patients with CC remain challenging. Notably, the survival rates for CC have seen limited improvement since the 1970s, particularly in low Human Development Index (HDI) countries, where the 5-year survival rate is < 20%, compared to high HDI countries with survival rates of 60–70% [2]. With the widespread application of bioinformatic analysis, numerous key genes related to CC prognosis have been identified, these genes have been explored from various angles, offering valuable insights for CC treatment [3–4]. Given the complex pathology of CC, comprehensive and in-depth research is still essential to enhance our understanding of its pathogenesis and molecular mechanisms, providing crucial targets for clinical research and treatment.

N6-methyladenosine (m6A) is the most prevalent RNA modification in eukaryotic, playing a crucial role in post-transcription and various biological processes and disease pathogenesis [5]. Ferroptosis is an iron-dependent cell death process characterized by lethal lipid peroxidation on the cell membrane [6]. In recent years, an inextricable connection between ferroptosis and m6A has emerged, affecting the occurrence and development of many tumors. For example, m6A in fibroblast growth factor receptor 4 (FGFR4), considered a risk factor for multiple cancer types, can attenuate ferroptosis in recalcitrant breast cancer [7, 8]. Furthermore, methyltransferase-like 3 (METTL3) can inhibit ferroptosis by modulating SLC7A11 m6A modification to promote lung adenocarcinoma tumor growth [8, 9]. However, no relevant studies have explored the mechanisms of m6A and ferroptosis in CC. Therefore, the present study aims to systematically analyze m6A-related ferroptosis genes (MRFGs) in CC and investigate the prognostic significance of these genes.

In this study, six differentially expressed MRFGs (DE-MRFGs) were identified and defined as biomarkers by analyzing CC data from the The Cancer Genome Atlas (TCGA) and The Gene Expression Omnibus (GEO) databases using bioinformatics and machine learning techniques. Subsequently, gene set enrichment analysis (GSEA), immune infiltration analysis, and survival analysis were performed to explore the impact of these six biomarkers. Furthermore, a competing endogenous RNA (ceRNA) network associated with these biomarkers was constructed to predict molecular regulatory mechanisms related to m6A and ferroptosis. Finally, biomarkers expression were verified by real-time quantitative polymerase chain reaction (RT-qPCR). This study is expected to provide new insights into the underlying factors of m6A-associated ferroptosis in CC and to have implications for the diagnosis, treatment, and prognosis of patients with CC.

## Materials and methods

### Data source

Two CC datasets, GSE9750 and GSE63514, were retrieved from the GEO database (https://www.ncbi.nlm.nih.gov/geo/). TCGA-CESC was obtained from the UCSC Xena (https://xenabrowser.net/datapages/). The GSE9750 dataset encompasses microarray sequencing data from 33 CC samples and 24 normal cervical epithelium samples generated using the GPL96 Affymetrix Human Genome U133A Array platform [10]. The GSE63514 dataset includes microarray sequencing data of 28 CC samples and 24 normal samples sequenced using the GPL570 Affymetrix Human Genome U133 Plus 2.0 Array platform [11]. The GSE7803 dataset includes microarray sequencing data of 21 CC samples and 10 normal samples sequenced using the GPL96 Affymetrix Human Genome HG-U133A Array platform [12]. The TCGA-CESC dataset comprises 309 CC samples, of which 296 samples, designated with “01A” to denote primary solid tumor samples without formalin immersion, were included in this study. Furthermore, a total of 23 m6A RNA methylation-related genes were derived from a publication [13], and 274 ferroptosis-related genes were sourced from the FerrDb database (http://www.zhounan.org/ferrdb/current/).

### Identification of DE-MRFGs

To begin, the “limma” R package (version 3.52.4) [14] was employed to detect differentially expressed genes (DEGs) between CC and control samples using the expression data from the GSE9750 dataset. The cut-off criteria for selection included a *p.adjust*-value < 0.05 and|log_2_FC| > 1. Subsequently, the DEGs were visualized through a volcano map and heatmap using the “ggplot2” R package (version 3.3.6) [15] and the “pheatmap” R package (version 1.0.12) [16], respectively. Following this, m6A and ferroptosis-related genes were matched with the GSE9750 dataset. MRFGs were then determined between m6A and ferroptosis-related genes via Spearman correlation analysis, conducted using the “psych” R package (version 2.2.9) [17]. The cut-off criteria involved a *p*-value < 0.05 and|cor| > 0.5. Finally, the DEGs were intersected with MRFGs using the “ggvenn” R package (version 0.1.9) [18] to yield DE-MRFGs.

### Enrichment and somatic mutation analysis of DE-MRFGs

The common functions of DE-MRFGs were identified through Gene Ontology (GO) and the Kyoto Encyclopedia of Genes and Genomes (KEGG) analyses, executed using the “clusterProfiler” R package (version 4.7.1.001) [19]. The outcomes of the GO and KEGG analyses were illustrated using a GOCircle plot and an enrichment map, generated with the “GOplot” R package (version 1.0.2) [20] and the “enrichplot” R package (version 1.16.2) [21], respectively. Considering that somatic mutations can be passed on to descendant cells during cell division, potentially playing a pivotal role in tumor development, we utilized the “maftools” R package (version 2.12.0) [22] to extract information on somatic mutations within DE-MRFGs from CC samples in the TCGA-CESC dataset (comprising 289 samples with somatic mutation data). The results were then visually presented.

### Recognition and verification of m6A-related ferroptosis biomarkers

The identification of biomarkers from the DE-MRFGs involved the utilization of the least absolute shrinkage and selection operator (LASSO) regression and support vector machine recursive feature elimination (SVM-RFE) algorithms based on the expression data from the GSE9750 dataset. These methods were implemented using the “glmnet” R package (version 4.1-6) [23] and “e1071” R package (version 1.7–12) [24], respectively. Common feature genes were derived from the intersection of these two algorithms using the “eulerr” R package (version 7.0.0) [25]. Subsequently, receiver operating characteristic (ROC) analysis was conducted to obtain the biomarkers, utilizing data from the GSE9750 and GSE63514 datasets. This analysis was performed using the “pROC” R package (version 1.18.0) [26]. Moreover, Kaplan–Meier (KM) survival analyses were employed to explore the association between these biomarkers and the survival status of CC samples in the TCGA-CESC dataset (comprising 283 samples with survival data). The “survminer” R package (version 0.4.9) [27] was employed for this purpose. CC samples were categorized into high-expression and low-expression groups based on the optimal threshold of biomarker expression. KM survival curves and log-rank tests were used to assess differences in survival status between these groups. In addition, the expression levels of the biomarkers in CC and control samples were evaluated in the GSE9750 and GSE63514 datasets using the Wilcoxon test. Finally, based on the GSE7803 dataset, we performed ROC analysis and expression validation of the biomarkers.

### Construction and verification of nomogram

To assess the utility of the biomarkers, a nomogram was created by incorporating the selected biomarkers. Scores based on the expression of each biomarker in the GSE9750 dataset were generated using the “rms” R package (version 6.3-0) [28]. The nomogram, integrated with the biomarkers, was established and subsequently validated through calibration curves.

### Gene set enrichment analysis (GSEA) of biomarkers

GSEA was performed to investigate the functional associations of the biomarkers using the GSE9750 dataset. The correlation between the biomarkers and all the genes in the GSE9750 dataset was assessed using the Spearman correlation analysis through the “psych” R package. The GSEA employed the “clusterProfiler” R package and GSEA gene sets obtained from the GSEA website (http://www.gsea-CCigdb.org/gsea/CCigdb). The *p*-values were sorted in ascending order, and the top 5 pathways were visualized in the enrichment map.

### Correlation analysis of immune infiltration and biomarkers

Given the reported associations among m6A, ferroptosis, and immune responses, single-sample GSEA (ssGSEA) was employed to identify differences in immune cells between CC and control samples, using expression data from GSE9750. Immune cell scores for each sample were computed using the “GSVA” R package (version 1.44.5) [29], and the distribution of immune cell scores was visualized in a heatmap generated with the “pheatmap” R package. Significantly different immune cell scores (SDICS) between CC and control samples were identified using the Wilcoxon test. The correlation between biomarkers and SDICS was assessed using the Spearman correlation analysis, and the results were visualized using the “ggcor” R package (version 0.9.8.1) [30].

### Correlation between biomarkers and oncogenic pathways

To delve deeper into the connection between biomarkers and oncogenic pathways, 13 cancer-related signatures (CRS) were acquired from a relevant publication [31]. CRS scores for each sample within the GSE9750 dataset were computed using the ssGSEA algorithm in the “GSVA” R package. The correlation between biomarkers and CRS was explored using the Spearman correlation analysis, and the results were visualized using the “ggcor” R package. Subsequently, the Kruskal test was used to identify differentially expressed biomarkers across various clinicopathological characteristics, including pathological M, N, and T stages, Stage, and Grade.

### Competing endogenous RNAs (ceRNA) network of biomarkers

To investigate the potential ceRNAs interacting with the biomarkers, a ceRNA network was constructed. Initially, starBase and miRTarBase databases were employed to predict the miRNAs associated with the biomarkers. Common miRNAs identified from both databases were used to predict the interacting lncRNAs separately in miRNet and starBase databases, respectively. The common lncRNAs were obtained from the intersection of lncRNAs from the miRNet and starBase databases. The cut-off criteria were clipExpNum > 10 in starBase and a total number of experimental data > 1 in miRTarBase. All lncRNAs predicted by miRNet were included. The common lncRNAs, in combination with the common miRNAs, were used to establish the ceRNA network.

### Real-time quantitative polymerase chain reaction (RT-qPCR)

We collected 10 cervical tissue samples from the Second Affiliated Hospital of Guizhou University of Traditional Chinese Medicine, including 5 CC tissue samples and 5 control samples, for subsequent RT-qPCR experiments. The Ethics Committee at Guizhou University of Chinese Medicine gave its approval for this study (Approval number: LW20231123). In detail, total RNA was extracted from the tissue samples using TRIzol reagent according to the manufacturer’s instructions, followed by reverse transcription to obtain cDNA using the SweScript First Strand cDNA synthesis kit. Subsequently, 40 cycles of the reaction were performed on a CFX96 real-time quantitative fluorescence PCR instrument under the following conditions: 95 ℃ for 1 min, 95 ℃ for 20 s, 55 ℃ for 20 s, and 72 ℃ for 30s. The relative expression of each biomarker was calculated by quantitative analysis method using 2^-ΔΔCt^, and GAPDH was used as an internal reference gene. The primer sequences were listed in Table 1.

**Table 1:** The primer sequences of ALOX12, EZH2, CDCA3, CDC25A, HSPB1 and GAPDH

Gene	Forward Primer	Reverse Primer
*ALOX12*	TCTGGAGATGGCCCTCAAAC	GAAGCTCTTCCATCCCCGAG
*EZH2*	AGGACGGCTCCTCTAACCAT	AAGGGCACGAACTGTCACAA
*CDCA3*	CTGTCCCTCCCTTGGTTTGG	CTGATCCAGCCCACTTGTGT
*CDC25A*	GGTAAGAGGTGTAGGTCGGC	TGTCTTCGCTGTTCTCCCAC
*HSPB1*	GGAGTGGTCGCAGTGGTTAG	GGGAGATGTAGCCATGCTCG
*GAPDH*	CGAAGGTGGAGTCAACGGATTT	ATGGGTGGAATCATATTGGAAC

### Statistical analysis

All analyses were conducted using the R programming language. Unless otherwise specified, a *p*-value < 0.05 was considered statistically significant.

## Results

### Identification of DE-MRFGs in CC

**Fig. 1:**
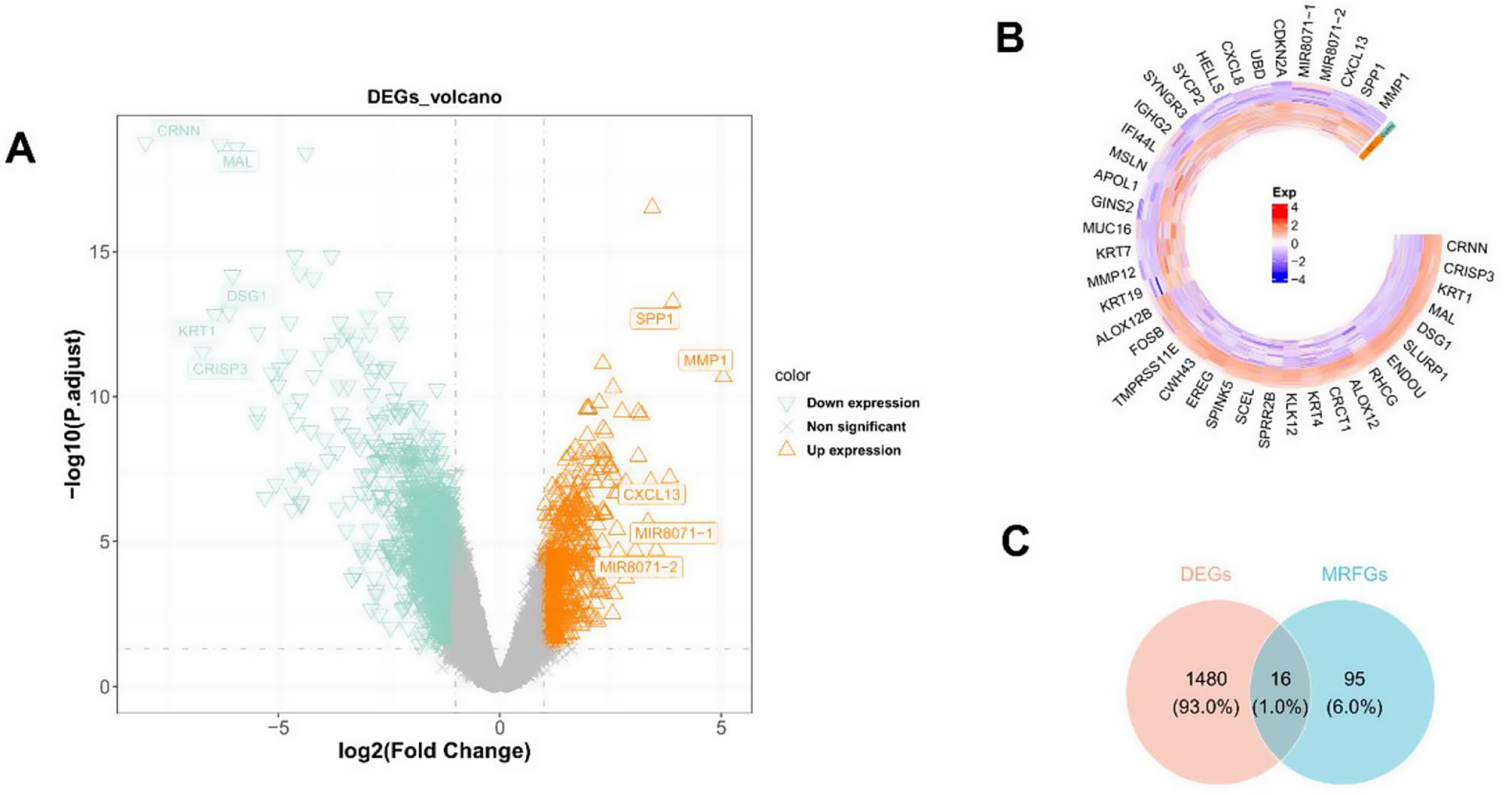
Identification of differentially expressed N6-methyladenosine (m6A)-related ferroptosis genes (DE-MRFGs) in cervical cancer (CC). (**A**) The volcano maps and (**B**) heatmap of differentially expressed genes (DEGs) between CC and control samples in the GSE9750 dataset. The orange equilateral triangle (red cell) and green inverted triangle (purple cell) represent upregulated and downregulated DEGs, respectively. (**C**) The Venn map of DE-MRFGs shared by DEGs and ferroptosis genes

A total of 1496 DEGs were identified between CC and control samples in the GSE9750 dataset, including 431 upregulated genes and 1065 downregulated genes (Supplementary Table 1, Fig. 1A). Figure 1B shows the expression of the top 40 DEGs in a heatmap. Subsequently, 18 m6A-related genes and 241 ferroptosis-related genes were matched within the GSE9750 dataset. Among these, 111 MRFGs were identified through the Spearman correlation analysis (Supplementary Table 2, Supplementary Fig. 1). As shown in Figs. 1C and 16 DE-MRFGs were observed, exhibiting differential expression in CC and control samples in the GSE9750 dataset.

### Functional enrichment and somatic mutation analyses of DE-MRFGs in CC

**Fig. 2:**
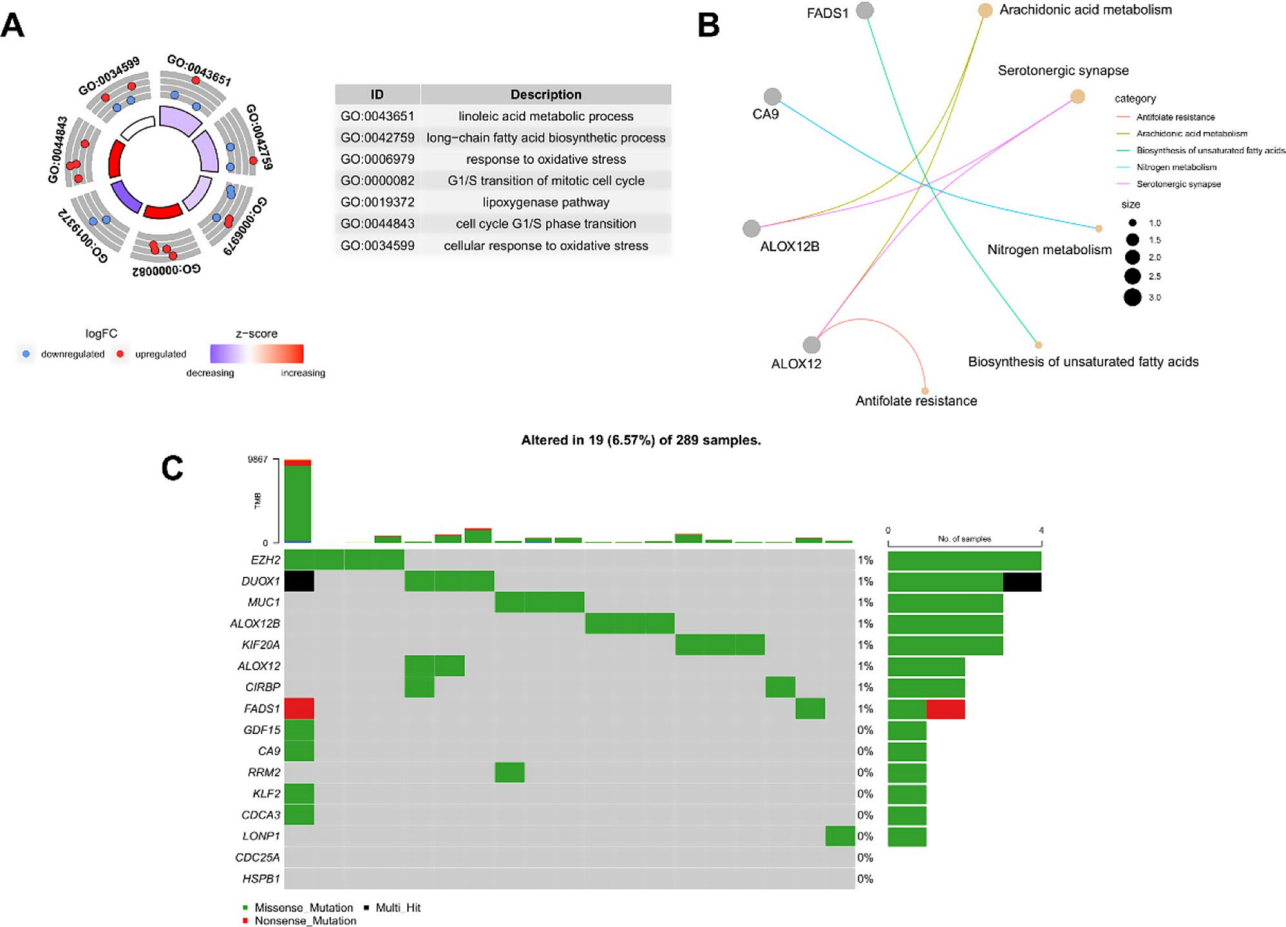
Functional enrichment and somatic mutation analyses of DE-MRFGs in CC. String diagram for Gene Ontology (GO) (**A**) and Kyoto Encyclopedia of Genes and Genomes (KEGG) (**B**) Enrichment analyses of DE-MRFGs. The outer circle is the pathway ID, the middle circle is the gene enriched in the term, and the inner circle is the z-score (which predicts whether the pathway is activated or suppressed). (**C**) Mutation waterfall of DE-MRFGs. The box plot in the top shows the tumor mutational burden (TMB) score of each sample and the waterfall shows the mutation of each gene (row) in each sample (column). Different colors represent different mutation types, and the bar chart on the right shows the proportion of samples with different mutation types in the gene

To elucidate the molecular mechanisms involving DE-MRFGs, GO and KEGG analyses were conducted. Figure 2A displays the top seven GO items, while Fig. 2B presents the top five KEGG pathway-enriched genes. Notably, DE-MRFGs were enriched in metabolism-related pathways, including linoleic acid metabolic process and arachidonic acid metabolism. Somatic mutation analysis was performed to explore the mutation status of DE-MRFGs in patients with CC. DE-MRFG mutations were detected in 19 out of 289 CC samples from the TCGA-CESC dataset. Most mutations were of the missense type. Among DE-MRFGs, *EZH2* and *DUOX1* mutations were observed in four CC samples, representing the highest number of samples affected (Fig. 2C).

### Identification of biomarkers in CC

**Fig. 3:**
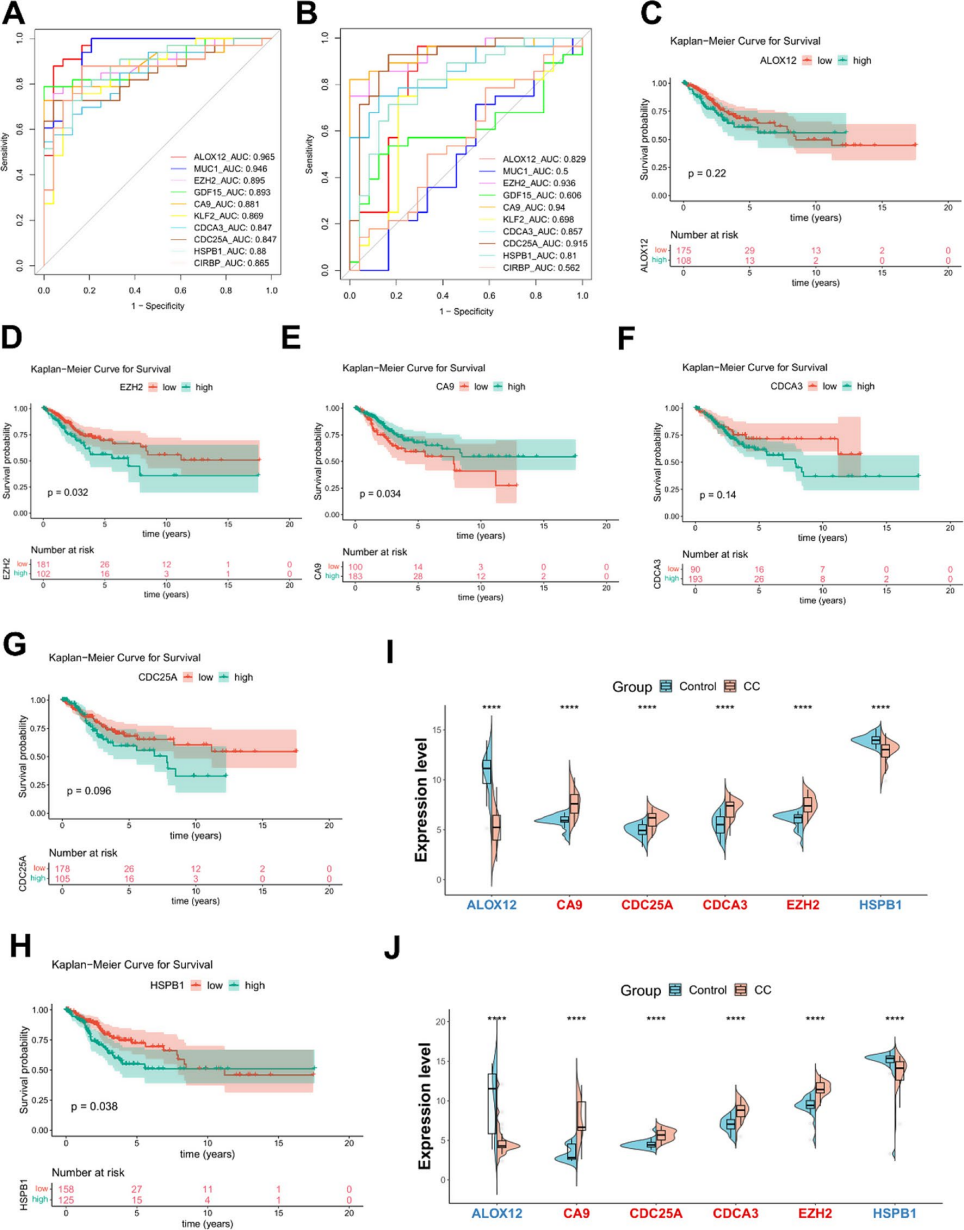
Identification of biomarkers in CC. Receiver Operating Characteristic (ROC) curves for 10 intersection genes in predicting the immunotherapy response in the GSE9750 (**A**) and GSE63514 (**B**) datasets. AUC: area under curve. Kaplan–Meier curves for the overall survival (OS) of patients in the high-expression and low-expression groups with biomarkers *ALOX12* (**C**), *EZH2* (**D**), *CA9* (**E**), *CA9* (**F**), *CDC25A* (**G**), and *HSPB1* (**H**) in the TCGA-CESC database. Expression of six feature genes in the CC and control samples in the GSE9750 (**I**) and GSE63514 (**J**) datasets

To identify biomarkers from the pool of DE-MRFGs, machine learning techniques were performed. Lasso regression selected 11 genes out of the 16 DE-MRFGs, including *ALOX12*,* MUC1*,* EZH2*,* GDF15*,* CA9*,* DUOX1*,* KLF2*,* CDCA3*,* CDC25A*,* HSPB1*, and *CIRBP*. SVM-RFE selected 13 genes from the 16 DE-MRFGs, including *HSPB1*,* EZH2*,* CIRBP*,* CA9*,* CDC25A*,* CDCA3*,* RRM2*,* ALOX12*,* GDF15*,* KLF2*,* MUC1*,* ALOX12B*, and *KIF20A*. A set of 10 common genes were identified through both algorithms, namely *ALOX12*,* MUC1*,* EZH2*,* GDF15*,* CA9*,* KLF2*,* CDCA3*,* CDC25A*,* HSPB1*, and *CIRBP*. To assess the diagnostic ability and generalizability of these 10 common genes, ROC analyses were conducted using the GSE9750 and GSE63514 datasets (Fig. 3A–B). Ultimately, six genes, including *ALOX12*,* EZH2*,* CA9*,* CDCA3*,* CDC25A*, and *HSPB1*, were recognized as biomarkers, exhibiting area under the curve (AUC) values > 0.8 in both GSE9750 and GSE63514 datasets. For the evaluation of the biomarkers’ potential impact on survival and prognosis, KM survival curves were generated (Fig. 3C–H). CC samples with available survival information were categorized into high-expression and low-expression groups based on the optimal threshold (minprop = 0.3) for the expression of the biomarkers. Notably, the survival status of *HSPB1*,* EZH2*, and *CA9* showed significant differences among these groups. Furthermore, the six biomarkers consistently demonstrated significantly differential expression in the training and testing datasets (Fig. 3I–J). In addition, the ROC results from the external validation dataset GSE7803 showed that the AUC values of these six biomarkers were greater than 0.7. The expression levels between the normal group and the CC group were consistent with those in the training and testing datasets, indicating that our findings have certain universality and generalizability (Supplementary Fig. 8).

### Construction of a CC nomogram

**Fig. 4:**
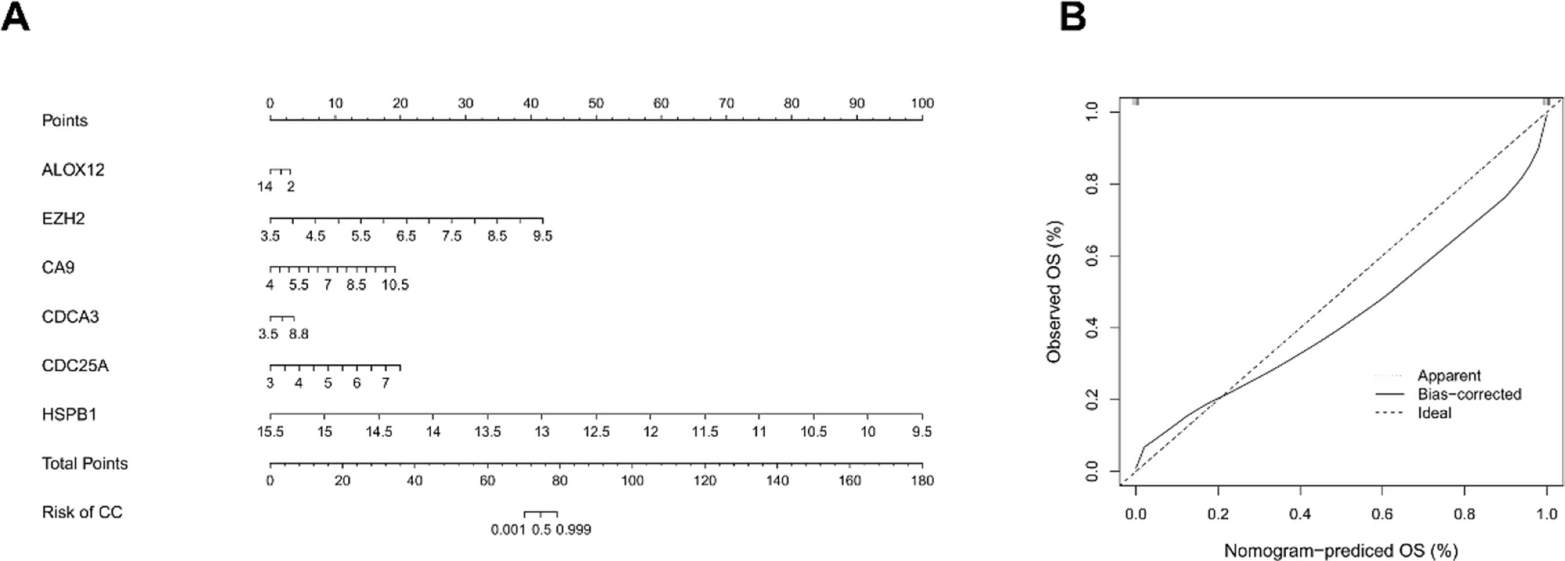
Construction and evaluation of a nomogram for CC. (**A**) Feature genes nomogram. The scores corresponding to each gene are added together to generate a total point. (**B**) Calibration curves of the nomogram. The horizontal and vertical coordinates represent the predicted probability and actual probability, respectively. The 45-degree line represents the ideal prediction

A nomogram was established using the six biomarkers (Fig. 4A). A higher total score on the nomogram corresponds to an increased likelihood of a CC diagnosis. The regression formula for the nomogram was computed as follows: y = 210.2324 + (− 0.3699) * *ALOX12* + (10.8376) * *EZH2* + (4.5802) * *CA9* + (1.0213) * *CDCA3* + (6.8987) * *CDC25A* + (− 25.9203) * *HSPB1*. Calibration curves confirmed the robust diagnostic accuracy of the nomogram (Fig. 4B).

### Enrichment analysis of biomarkers

**Fig. 5:**
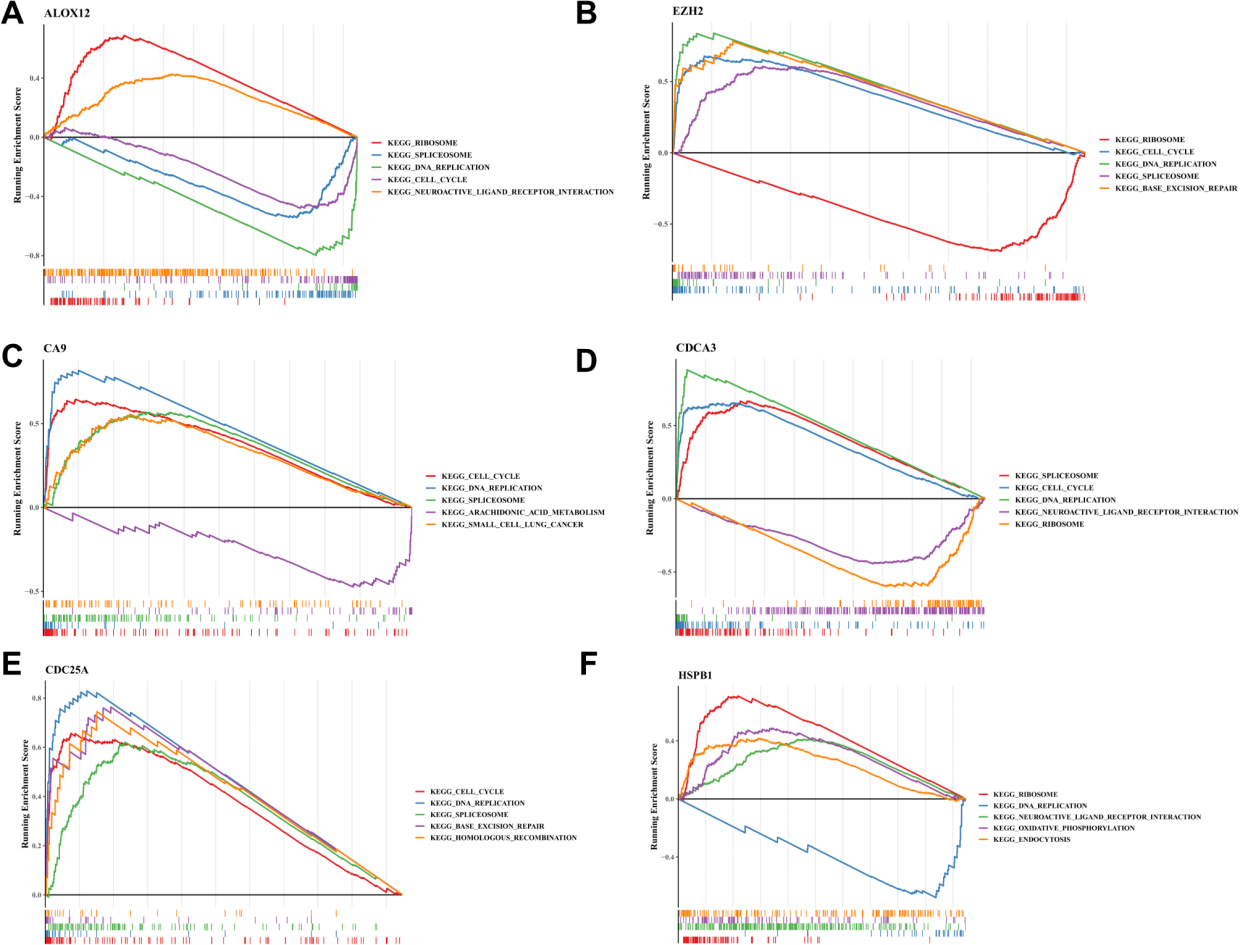
Gene Set Enrichment Analysis (GSEA) of biomarkers. (**A**) *ALOX12*. (**B**) *EZH2*. (**C**) *CA9*. (**D**) *CDCA3*. (**E**) *CDC25A*. (**F**) *HSPB1*. The horizontal axis represents the ranked gene according to the correlation coefficient with feature gene, and the vertical axis is the running enrichment score

To gain insights into the molecular mechanisms of the biomarkers, GSEA was conducted using KEGG gene sets, as shown in Fig. 5. *ALOX12* demonstrated connections with “ribosome”, “spliceosome”, “DNA replication”, “cell cycle”, and “neuroactive ligand–receptor interaction” (Fig. 5A). *EZH2* was linked to “ribosome”, “cell cycle”, “DNA replication”, “spliceosome”, and “base excision repair” (Fig. 5B). *CA9* exhibited correlations with “cell cycle”, “DNA replication”, “spliceosome”, “arachidonic acid metabolism”, and “small cell lung cancer” (Fig. 5C). *CDCA3* was associated with “spliceosome”, “cell cycle”, “DNA replication”, “neuroactive ligand–receptor interaction”, and “ribosome” (Fig. 5D). CDC25A showed connections to “cell cycle”, “DNA replication”, “spliceosome”, “base excision repair”, and “homologous recombination” (Fig. 5E). *HSPB1* was related to “ribosome”, “DNA replication”, “neuroactive ligand–receptor interaction”, “oxidative phosphorylation”, and “endocytosis” (Fig. 5F).

### Immune infiltration in CC

**Fig. 6:**
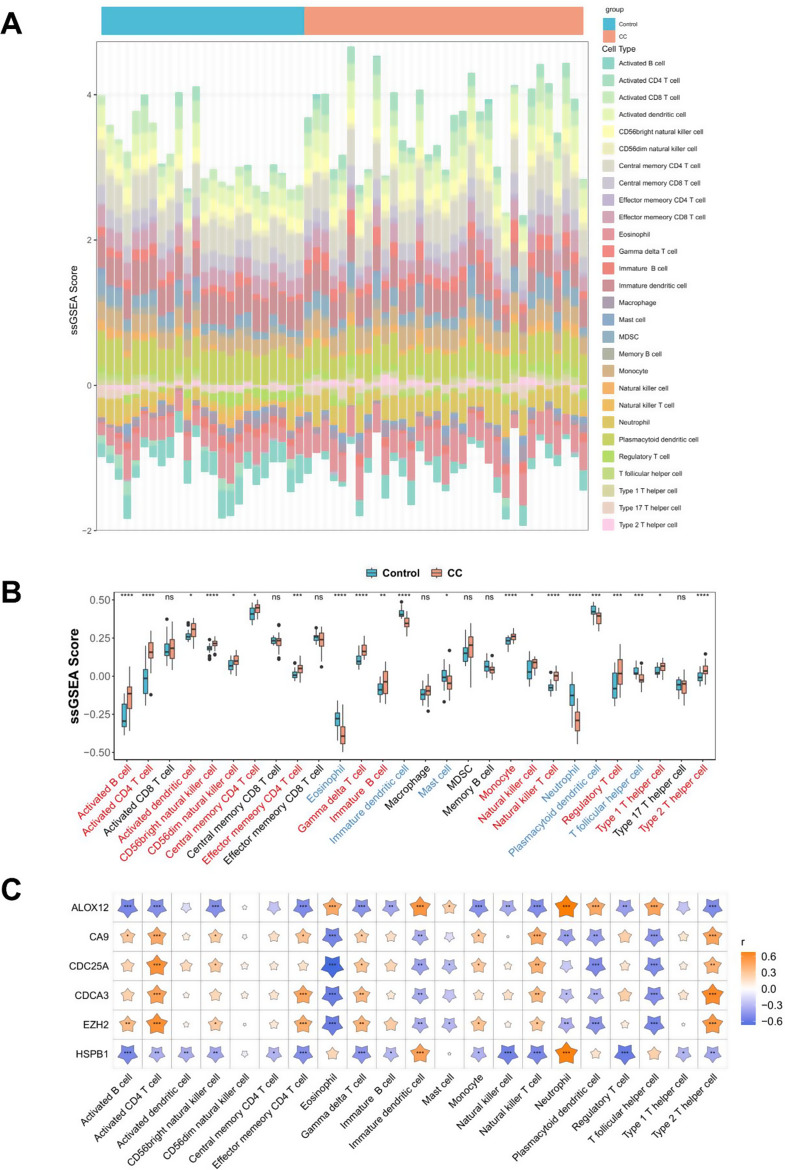
Immune infiltration in CC. (**A**) The heat maps of the scores of 28 immune cells in the GSE9750 dataset. (**B**) The box plots comparing immune cell scores between the control and CC samples. (**C**) Correlation matrix of biomarkers and immune cells. Shading color and asterisks represent the value of corresponding correlation coefficients. ns, not significant; *, *p* < 0.05; **, *p* < 0.01; ***, *p* < 0.001; ****, *p* < 0.0001

To explore immune cell infiltration, immune scores were calculated for each sample in the GSE9750 dataset using ssGSEA. As shown in Fig. 6A, the ssGSEA scores for 28 immune cells in the CC and control groups were presented in a heatmap. Notably, significant differences in ssGSEA scores were observed for 21 immune cells between the CC and control groups (Fig. 6B). Subsequent correlation analysis between immune cells and biomarkers revealed a robust relationship (Fig. 6C), with *ALOX12* demonstrating a positive association with neutrophils and *CDC25A* displaying a negative correlation with eosinophils.

### Oncogenic and clinical analysis of biomarkers

**Fig. 7:**
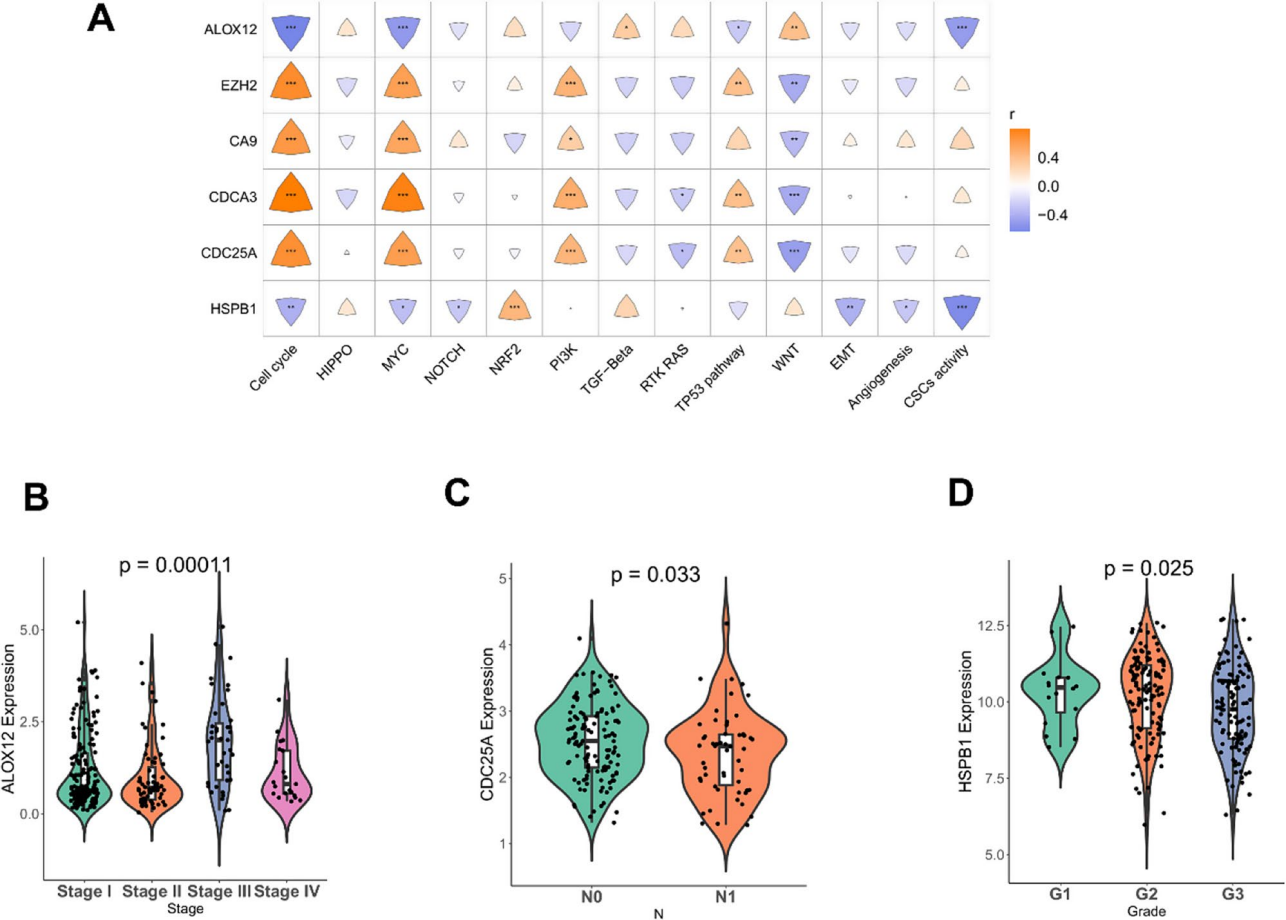
Oncogenic and clinical correlation analyses of biomarkers. (**A**) Heat map of the correlation between biomarkers and oncogenic pathways. The yellow indicates positive correlation and blue indicates negative correlation. Shading color and asterisks represent the value of corresponding correlation coefficients. ns, not significant; *, *p* < 0.05; **, *p* < 0.01; ***, *p* < 0.001; ****, *p* < 0.0001. (**B**) Expression of the *ALOX12* biomarker in Tumor stage. (**C**) Expression of the *CDC25A* biomarker in Pathological N stage. (**D**) Expression of the *HSPB1* biomarker in Grade

A total of 13 CRS were identified to explore the correlation between biomarkers and oncogenic pathways. *CDCA3* exhibited the highest positive correlation with “cell cycle”, while *ALOX12* displayed the highest negative correlation with “cell cycle” (Fig. 7A). Furthermore, differential expressions of the biomarkers were observed in various clinicopathological characteristics. For instance, *ALOX12* exhibited significantly different expression across different Stages (Fig. 7B). *CDC25A* demonstrated varying expression in different pathological N stages (Fig. 7C), and *HSPB1* displayed a distinct expression in different Grades (Fig. 7D). The expression of the remaining biomarkers did not show significant differences across various clinicopathological characteristics (Supplementary Figs. 2–7).

### CeRNA network of biomarkers

**Fig. 8:**
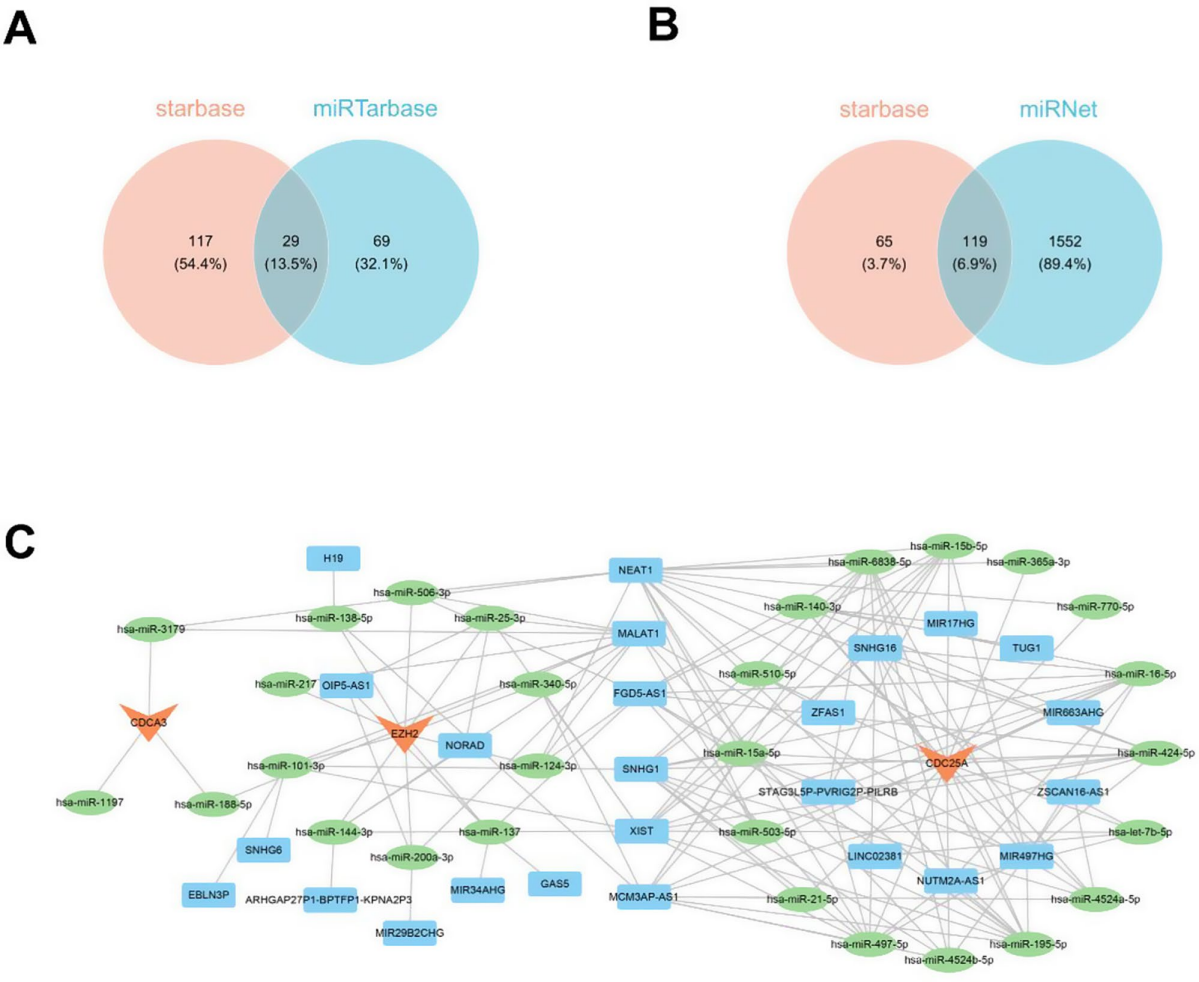
The competing endogenous RNA (ceRNA) network of biomarkers. (**A**) Venn map of microRNA (miRNA) targeting common biomarkers to the starBase and miRTarBase databases. (**B**) Venn map of long noncoding RNAs (lncRNA) targeting common miRNAs to the miRNet and starBase databases. (**C**) The ceRNA network. The red triangle is mRNA, the green circle is miRNA, and the blue rectangle is lncRNA

To investigate the ceRNA network of biomarkers, we employed predicted databases for miRNAs (starBase and miRTarBase) and lncRNAs (miRNet and starBase). A total of 29 miRNAs were predicted as the interacting miRNAs for the three biomarkers using starBase (Supplementary Table 3) and miRTarBase (Supplementary Table 4) databases (Fig. 8A, Supplementary Table 5). In addition, 25 lncRNAs (Supplementary Table 6) were identified as the interacting lncRNAs for 28 miRNAs in the miRNet (Supplementary Table 7) and starBase databases (Supplementary Table 8, Fig. 8B). The ceRNA network is depicted in Fig. 8C. For instance, *CDCA3* was regulated by hsa-miR-3179, hsa-miR-188-5p, and hsa-miR-1197. Furthermore, hsa-miR-3179 was regulated by lncRNAs NEAT1 and MALAT1. *CDC25A* was regulated by hsa-miR-424-5p, which, in turn, was regulated by lncRNAs STAG3L5P-PVRIG2P-PILRB, FGD5-AS1, SNHG16, SNHG1, NEAT1, XIST, MCM3AP-AS1, and MIR497HG.

### Validation of biomarkers through RT-qPCR

**Fig. 9:**
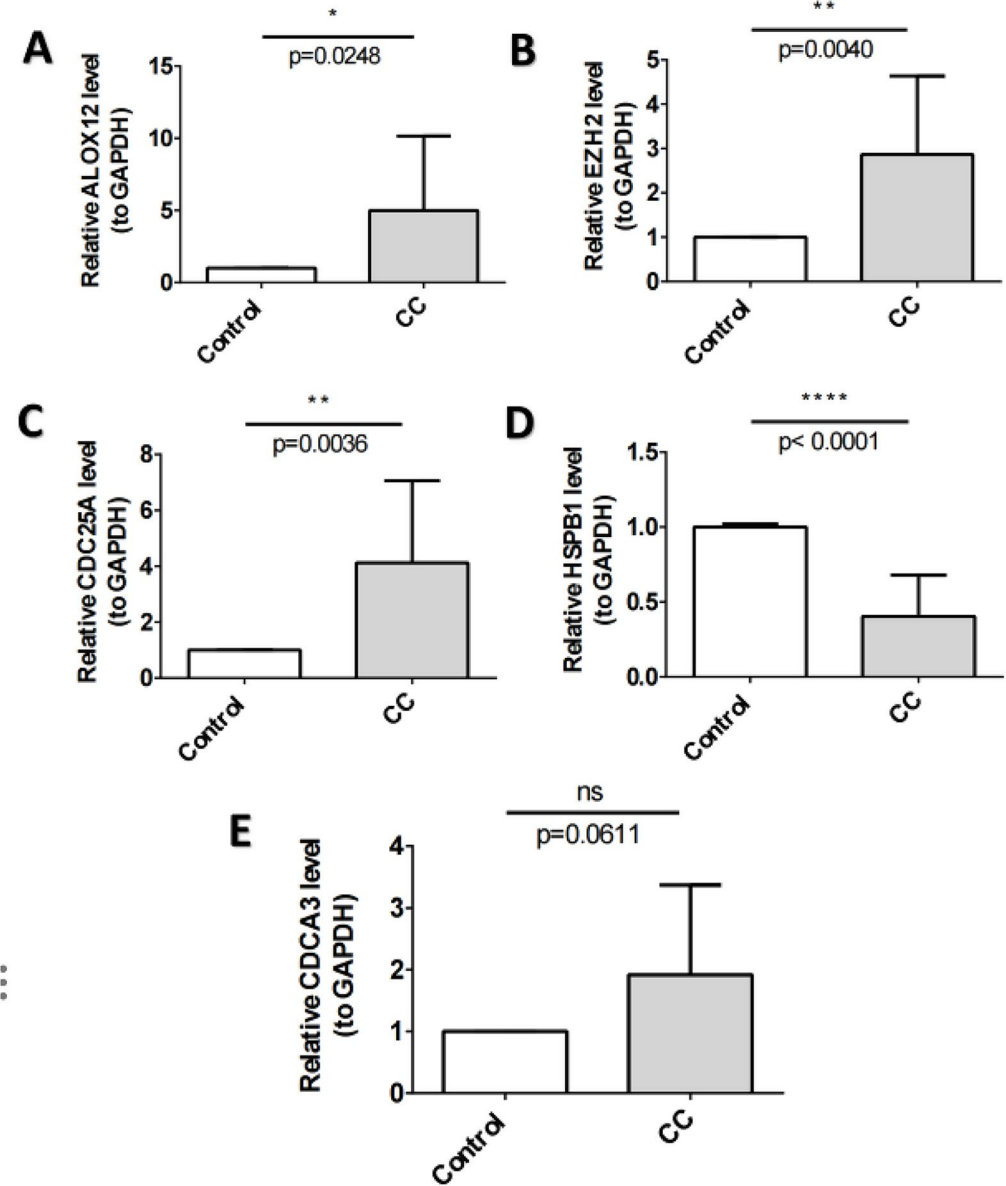
Validation of biomarkers through RT-qPCR. (**A**) Relative *ALOX12* level(to GAPDH). (**B**) Relative *EZH2* level(to GAPDH). (**C**) Relative *CDC25A* level(to GAPDH). (**D**) Relative *HSPB1* level(to GAPDH). (**E**) Relative *CDCA3* level(to GAPDH). The group of CC refers to the tumor samples, the group of control refers to peritumoral tumor samples. ns, not significant; *, *p* < 0.05; **, *p* < 0.01; ***, *p* < 0.001; ****, *p* < 0.0001

The RT-qPCR results showed that the expression levels of *ALOX12*, *EZH2*, and *CDC25A* were significantly elevated in the CC group, while the expression of *HSPB1* showed the opposite trend (*P* < 0.05) (Fig. 9A and D). We could observe that *CDCA3* expression was not significantly different between the two groups (Fig. 9E), probably due to the small sample size or sample heterogeneity.

## Discussion

Recent studies have demonstrated that m6A and ferroptosis can exert crucial influence over the specific biological processes in CC. METTL14 can reduce FTH1 mRNA stability through m6A methylation, thereby enhancing sorafenib-induced ferroptosis, which contributes to suppressing CC progression via the PI3K/Akt signaling pathway [32]. Yangmei Gong et al. demonstrated that miR-30c-5p repressed the growth and metastasis of CC xenografts through the inhibition of the METTL3/KRAS axis [33]. COTE-1 is a METTL3-mediated m6A modification target whose expression is dependent on the m6A reader. METTL3 promotes the proliferation and metastasis of CC cells and affects autophagy dependent iron death by regulating the expression of COTE-1 [34]. So we hypothesized that m6A might influence specific biological processes within CC tumors by affecting the RNA associated with ferroptosis. In the present study, we identified six biomarkers, including *ALOX12*,* EZH2*,* CA9*,* CDCA3*,* CDC25A*, and *HSPB1.* The construction of a nomogram revealed the strong predictive capabilities of these six biomarkers for CC, and their expression was validated in an external validation dataset and in samples we collected. Functional enrichment analysis using GO and KEGG indicated that these biomarkers were predominantly associated with oxidative stress and the cell cycle, among other factors. Furthermore, we observed associations among *EZH2*,* CA9*, and *HSPB1* with overall survival (OS) in CC, as well as relationships among *ALOX12*,* CDC25A*, and *HSPB1* with clinical progression in CC. Other bioinformatics studies have also identified connections among *EZH2*,* CA9*, and *HSPB1* with OS or clinical stages in CC, aligning with our findings [35–36].

In this study, the expression of *EZH2* in CC was significantly higher than in normal samples. EZH2 protein, a core subunit of Polycomb repressive complex 2, has been identified as being crucial for epigenetic modifications. Its histone methyltransferase activity leads to the methylation of target genes, influencing gene expression and impacting the survival status of various cancers [37]. Study had demonstrated that overexpression of EZH2 was observed in CC tissues and cervical intraepithelial lesions (CIN) tissues compared with paracancer normal tissues, and was closely correlated with tumor grade, histological differentiation, lymphatic metastasis, and overall survival. The strong upregulation of EZH2 showed strong diagnostic power in distinguishing CC and CIN tissues from normal tissues [38]. Thus, it is plausible that *EZH2* might similarly drive CC progression through mechanisms involving m6A and ferroptosis. Furthermore, the high expression of EZH2 combined with the inactivation of p53/p21 induces the cell to enter mitosis during the S phase. This dysregulation of the cell cycle may accelerate cancer cell death while enhancing chemotherapy efficacy, the role of EZH2 in cell cycle regulation, and its synergistic effect with WEE1 make this combination therapy strategy with great clinical potential [39].

*CA9*, also known as *CAIX*, plays a pivotal role in the regulation of acid-base balance and the transport of carbon dioxide and bicarbonate, ultimately influencing intracellular pH and extracellular pH balance. It is particularly significant in the context of anoxic and acidic TMEs. As a prominent hypoxia-inducing marker, *CA9* is known to be overexpressed in the majority of hypoxic solid tumor cells, contributing to tumor metastasis and poor prognoses [40]. This aligns with the findings of our study. A study by Jakubicková L et al. demonstrated that methylation of a specific CpG site at the − 74 position of the transcription start site leads to reduced *CA9* expression in high-density cultured CC HeLa cells [41]. Serum CA9 level plays an important role in the prognosis assessment of patients with metastatic clear cell renal cell carcinoma (RCC) [42]. CA9 is associated with longer overall survival in acute myeloid leukemia (AML), and its high expression induces a strong immune response that helps control minimal residual disease (MRD). Therefore, CA9, as an immunotherapy target, may play an important role in the clinical treatment of AML, especially in multivalent immunotherapy [43].

In this study, *HSPB1* expression in CC tumors was lower than in normal samples, suggesting its protective function. *HSPB1*, also known as *HSP27*, belongs to the family of stress-inducible proteins, commonly referred to as heat shock proteins. It plays a crucial role in maintaining cellular homeostasis in normal cells. However, Alvarez-Olmedo DG et al. discovered that *HSPB1* could protect CC cells from oxidative and toxic damage induced by cadmium [44]. *HSPB1* overexpression can inhibit ferroptosis in CC cells induced by erastin [45]. These findings seemingly contrast the conclusions we reached, necessitating further investigation. The promoter − 1271G > C variant of the *HSPB1* gene is closely associated with lung cancer susceptibility and survival. Patients with the − 1271 C allele have an increased risk of lung cancer, but offer better survival in patients with advanced non-small cell lung cancer (NSCLC). This variant was also associated with levels of DNA damage and reduced expression of Hsp27. In conclusion, the − 1271G > C variant may affect the susceptibility and prognosis of lung cancer by regulating the level of Hsp27 synthesis, which has important clinical value [46].

*CDC25A* is among the most critical regulators of the cell cycle. Qi J et al. indicated that histone demethylation of *CDC25A* can result in DNA instability and promote CC progression [47]. In addition, *CDC25A* has been found to suppress autophagy-mediated ferroptosis in CC [48]. In this study, higher *CDC25A* expression was observed in CC compared to healthy individuals, so it is plausible that *CDC25A* is involved in CC progression through the mechanism of m6A and ferroptosis. CDC25A may have important clinical value in epithelial tumors because LIN28A regulates its expression by inhibiting let-7 miRNA biogenesis. The upregulation of CDC25A is able to promote cell cycle progression in cancer cells, and therefore, as a cell cycle regulator, it could be a potential target in cancer therapy, especially in tumor types where LIN28A is reactivated [49].

*ALOX12* is a member of the lipoxygenase family, responsible for oxidizing unsaturated fatty acids to produce lipid peroxides and bioactive lipids. This function allows it to play a role in regulating cell proliferation, apoptosis, differentiation, and senescence [50]. In this study, decreased *ALOX12* expression was observed in CC tumor tissues, suggesting its protective role. A study showed that down-regulation of *ALOX12* can inhibit ferroptosis induced by cancer suppressor pathway p53 and accelerate tumor formation [51]. However, studies in other cancers have reported different outcomes. For instance, reduced *ALOX12* expression can increase sensitivity to chemotherapy in breast cancer and inhibit the EMT, suppressing progression in lung cancer [52]. Genetic variations in the *ALOX12* gene were strongly associated with adenoma recurrence, especially in the patient population taking placebo. Its variation may be associated with adenoma recurrence and cardiovascular toxicity caused by celecoxib therapy [53]. Further studies found that abnormal methylation of the *ALOX12* gene was associated with megakaryocyte dysplasia in acute myeloid leukemia (AML) (*P* = 0.0003) and poorer prognosis (overall survival and disease-free survival, *P* = 0.000411). Therefore, this gene has clinical application value in predicting the prognosis of patients and guiding individualized treatment [54]. However, clinical studies on ALOX12 in CC have not yet been reported, so further clinical studies are needed to explore the potential role of ALOX12 in the occurrence, development and treatment response of cervical cancer.

*CDCA3* is a gene associated with cell cycle progression, and our results showed an up-regulated expression of *CDCA3* in CC. In gastric cancer, the CDCA3 protein can promote the transition of the cell cycle from the G0/G1 phase to the S phase, thereby stimulating tumor proliferation [55]. However, Katrina Kildey et al. found that *CDCA3* can enhance the therapeutic efficacy of platinum-based chemotherapy [56]. So far, there isn’t any experiment demonstrated that *CDCA3* is associated with m6A or ferroptosis in CC, so it is worthy of further investigation.

In this study, *ALOX12* was found to have associations with various immune cell infiltrations, with the highest positive correlation observed with neutrophils. Although not previously reported in CC, Siyuan Weng et al. identified that high *ALOX12* expression predicted increased immune infiltration and improved immunotherapy response in colon cancer [57]. Consequently, further research in CC regarding the potential immunomodulatory role of *ALOX12* is warranted. In addition, we found that *CDC25A* was linked to multiple immune cell infiltrations and had the strongest negative correlation with eosinophils. Two other bioinformatic analyses have demonstrated the influence of *CDC25A* on immune cell infiltration and tumor development in lung adenocarcinoma and liver hepatocellular carcinoma [58–59]. Hence, it is essential to conduct further investigations to understand how *CDC25A* impacts immune cell infiltration in CC and its consequent effects on CC progression. Moreover, we observed that four other biomarkers were associated with various immune cell infiltrations, including “activated CD4 T cell”, “effector memory CD4 T cell”, and “eosinophil”. A study showed that *EZH2* plays a central role in regulating T-cell immune responses and regenerating chimeric antigen receptor T cells, and it can remodel the TME in several solid tumors [60]. *CA9* acts as an immunoadjuvant, stimulating adaptive immune responses against tumor antigens. In pancreatic cancer, CA9-mediated acidic TME suppresses immune infiltration of CD8 + T cells [61]. *HSPB1* can modulate immune escape in ovarian cancer, and inhibiting *HSPB1* inhibition can enhance killing and memory responses mediated by CD8 + T cells in breast cancer [62–63]. A study indicated that *CDCA3* can serve as a prognostic biomarker in cutaneous melanoma and is associated with immune infiltration [64]. Moreover, in renal cell carcinoma, *CDCA3* can predict a poor prognosis and impact CD8 + T cell infiltration [65]. In summary, these biomarkers play significant roles in tumor occurrence and development by positively or negatively affecting immune cell infiltration, thereby influencing the TME. This suggests that further investigations into these biomarkers in CC are warranted.

Lastly, we predicted miRNAs and lncRNAs associated with the six biomarkers and constructed a ceRNA network to identify potential regulatory interactions. The ceRNA network consisted of three biomarkers (*CDC25A*,* CDCA3*, and *EZH2*), 29 miRNAs, and 25 lncRNAs. According to a previous study, several miRNAs and their related lncRNAs can regulate these three biomarkers and influence the progression of various cancers. For instance, LINC01535 can suppress miR-214 expression, resulting in increased *EZH2* expression and contributing to the poor prognosis of CC [66]. Wei J et al. demonstrated that LINC00662 facilitates the progression of CC and the development of radiotherapy resistance through the absorption of microRNA-497-5p, which consequently results in the indirect up-regulation of CDC25A expression [67]. The lncRNA ST8SIA6-AS1 inhibits the p53/p21 pathway through the targeted inhibition of miR-145-5p/*CDCA3*, consequently promoting the proliferation and metastasis of breast cancer cells [68]. In this study, we identified several miRNAs and their associated lncRNAs for the three biomarkers that have not been previously reported in published studies. These findings represent potential novel mechanisms underlying CC that merit further investigation.

In conclusion, we initially identified six ferroptosis-associated m6A biomarkers in CC using various bioinformatic analyses. Biomarkers can help detect diseases that are not obvious or difficult to diagnose, assess a patient’s risk of disease, and take early intervention measures [69]. Meanwhile, these biomarkers offer potential diagnostic and therapeutic insights for CC and serve as a foundation for further molecular research. Functional, immune infiltration, and ceRNA analyses suggest the involvement of these biomarkers in CC development. However, clinical application of these findings necessitates additional data from larger sample sizes, and the specific mechanisms through which these biomarkers operate require further experimental validation. This study also lacks additional, larger, independent data sets for external validation to improve the reliability and generalisability of our study. Therefore, we plan to confirm and extend the findings with a larger clinical cohort through external validation or multi-center collaboration in future studies. Our ongoing efforts aim to elucidate the precise roles of these biomarkers in CC.

## Electronic supplementary material

Below is the link to the electronic supplementary material.

Supplementary Material 1: Figure 1. Sankey diagram of the interaction between N6-methyladenosine (m6A)-related genes and m6A-related ferroptosis genes (MRFGs). Left: m6A; right: MRFGs. Figure 2. Presentation of the *ALOX12* expressions in various clinical subgroups (*p* > 0.05). Figure 3. Presentation of the *CA9* expressions in various clinical subgroups (*p* > 0.05). Figure 4. Presentation of the *CDC25A* expressions in various clinical subgroups (*p* > 0.05). Figure 5. Presentation of the *CDCA3* expressions in various clinical subgroups (*p* > 0.05). Figure 6. Presentation of the *EZH2* expressions in various clinical subgroups (*p* > 0.05). Figure 7. Presentation of the *HSPB1* expressions in various clinical subgroups (*p* > 0.05). Figure 8. Based on the GSE7803 dataset, the ROC analysis and expression validation of the biomarkers. A: ROC. B: The expression of the biomarkers.

Supplementary Material 2: Table 1. Differentially expressed genes (DEGs) in cervical cancer (CC) and control samples in the GSE9750 dataset. Table 2. m6A-related genes and ferroptosis-related genes in the GSE9750 dataset. Table 3. miRNAs corresponding to 4 biomarkers in starBase database. Table 4. miRNAs corresponding to 4 biomarkers in miRTarBase database. Table 5. miRNAs corresponding to 3 biomarkers in starBase and miRTarBase database. Table 6. lncRNAs corresponding to miRNAs in miRNet and starBase database. Table 7. lncRNAs corresponding to miRNAs in miRNet database. Table 8. lncRNAs corresponding to miRNAs in starBase database.

## Data Availability

Sequence data that support the findings of this study have been deposited in GEO database (https://www.ncbi.nlm.nih.gov/geo/) and UCSC Xena (https://xenabrowser.net/datapages/).
