# Supplementary figures and images for: Identification of N6-methyladenosine-associated ferroptosis biomarkers in cervical cancer
Source: Hereditas. 2025 Apr 7;162:53. doi: 10.1186/s41065-025-00418-3 (PMC11974235; doi:10.1186/s41065-025-00418-3)

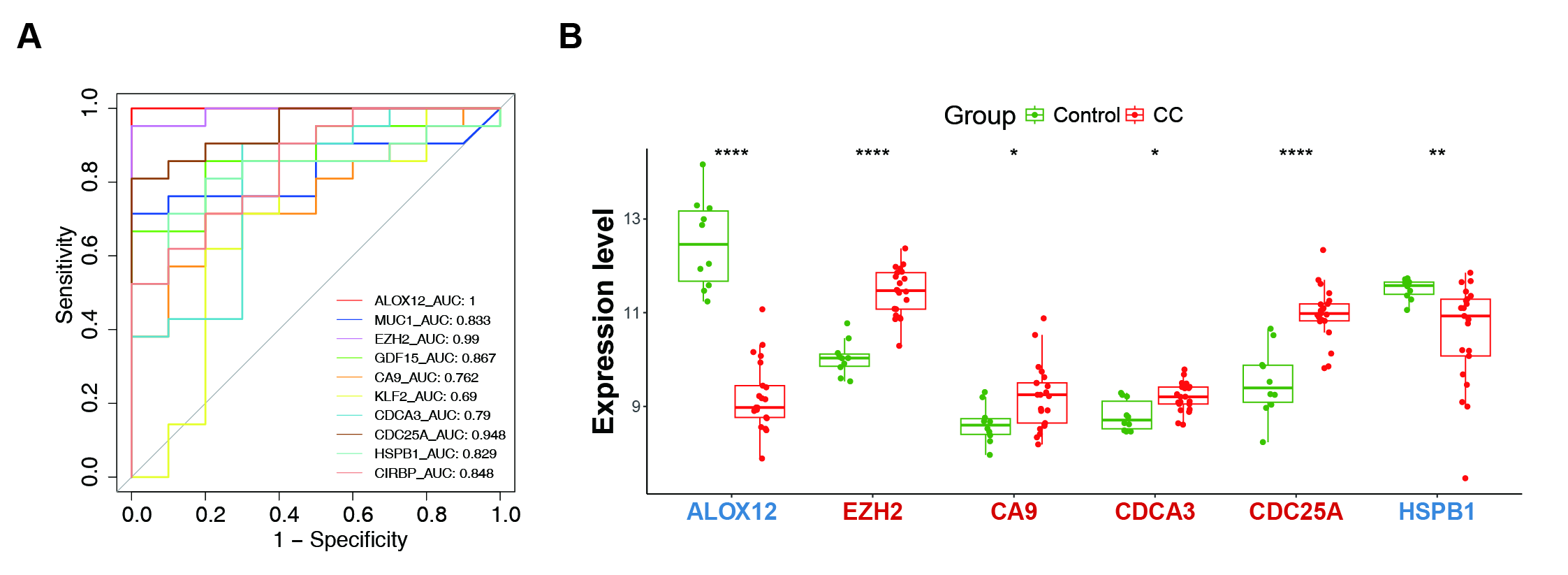

Supplement: Supplementary file 1 — Supplementary Material 1: Figure 1. Sankey diagram of the interaction between N6-methyladenosine (m6A)-related genes and m6A-related ferroptosis genes (MRFGs). Left: m6A; right: MRFGs. Figure 2. Presentation of the ALOX12 expressions in various clinical subgroups (p > 0.05). Figure 3. Presentation of the CA9 expressions in various clinical subgroups (p > 0.05). Figure 4. Presentation of the CDC25A expressions in various clinical subgroups (p > 0.05). Figure 5. Presentation of the CDCA3 expressions in various clinical subgroups (p > 0.05). Figure 6. Presentation of the EZH2 expressions in various clinical subgroups (p > 0.05). Figure 7. Presentation of the HSPB1 expressions in various clinical subgroups (p > 0.05). Figure 8. Based on the GSE7803 dataset, the ROC analysis and expression validation of the biomarkers. A: ROC. B: The expression of the biomarkers. [file 41065_2025_418_MOESM1_ESM.zip › Supplementary Figures/Supplementary Figure 8.tif]

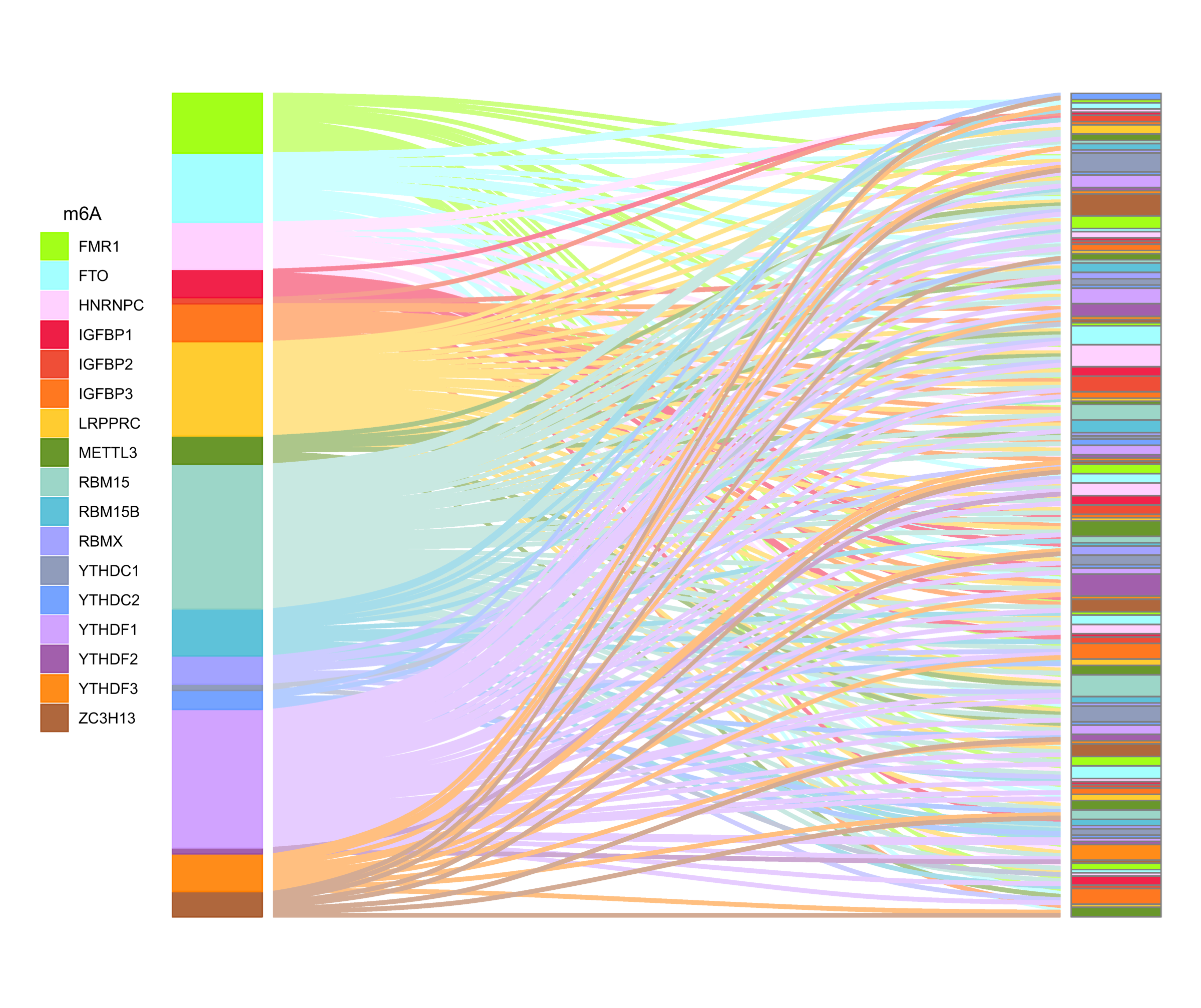

Supplement: Supplementary file 1 — Supplementary Material 1: Figure 1. Sankey diagram of the interaction between N6-methyladenosine (m6A)-related genes and m6A-related ferroptosis genes (MRFGs). Left: m6A; right: MRFGs. Figure 2. Presentation of the ALOX12 expressions in various clinical subgroups (p > 0.05). Figure 3. Presentation of the CA9 expressions in various clinical subgroups (p > 0.05). Figure 4. Presentation of the CDC25A expressions in various clinical subgroups (p > 0.05). Figure 5. Presentation of the CDCA3 expressions in various clinical subgroups (p > 0.05). Figure 6. Presentation of the EZH2 expressions in various clinical subgroups (p > 0.05). Figure 7. Presentation of the HSPB1 expressions in various clinical subgroups (p > 0.05). Figure 8. Based on the GSE7803 dataset, the ROC analysis and expression validation of the biomarkers. A: ROC. B: The expression of the biomarkers. [file 41065_2025_418_MOESM1_ESM.zip › Supplementary Figures/Supplementary Figure1.tif]

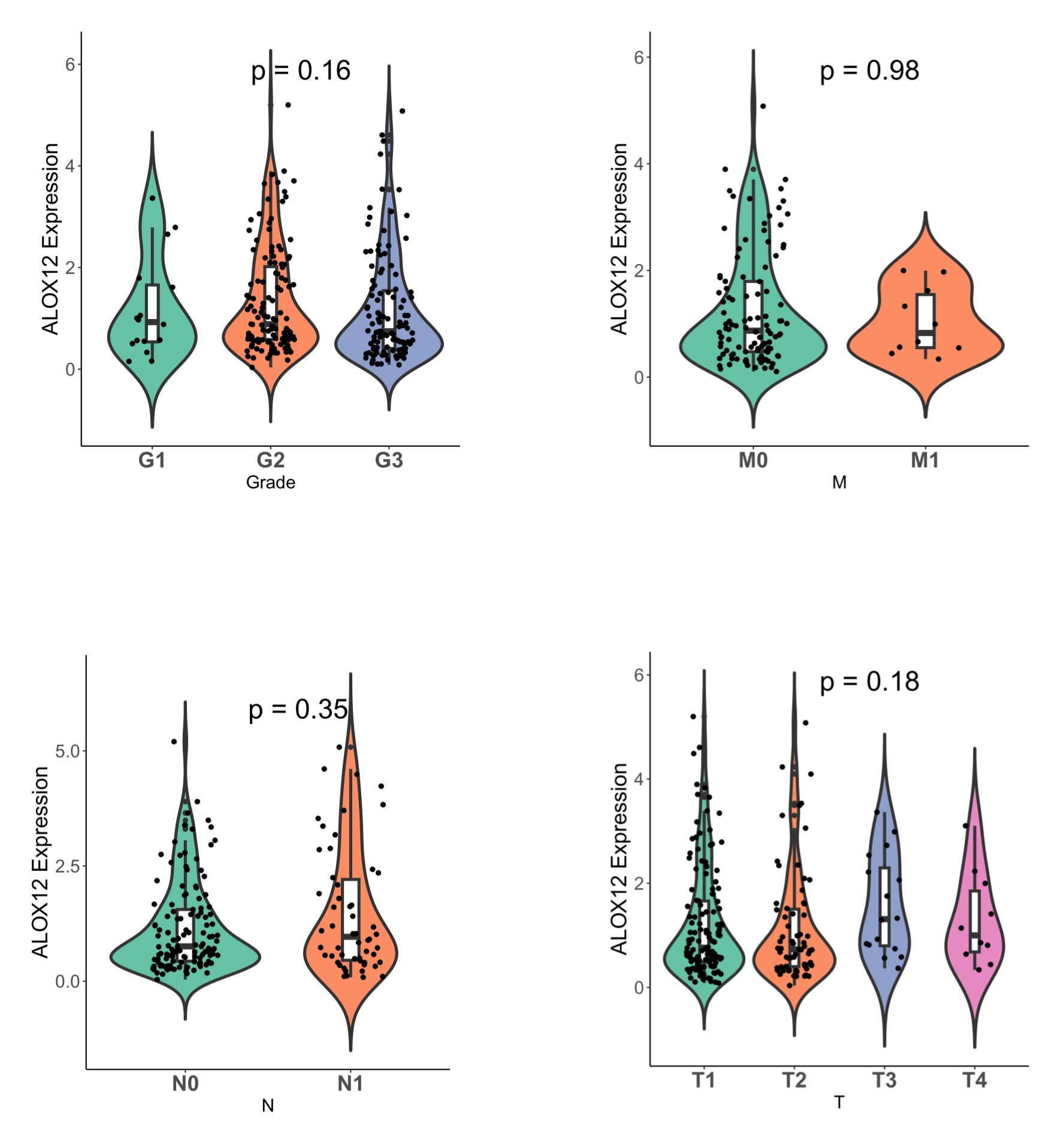

Supplement: Supplementary file 1 — Supplementary Material 1: Figure 1. Sankey diagram of the interaction between N6-methyladenosine (m6A)-related genes and m6A-related ferroptosis genes (MRFGs). Left: m6A; right: MRFGs. Figure 2. Presentation of the ALOX12 expressions in various clinical subgroups (p > 0.05). Figure 3. Presentation of the CA9 expressions in various clinical subgroups (p > 0.05). Figure 4. Presentation of the CDC25A expressions in various clinical subgroups (p > 0.05). Figure 5. Presentation of the CDCA3 expressions in various clinical subgroups (p > 0.05). Figure 6. Presentation of the EZH2 expressions in various clinical subgroups (p > 0.05). Figure 7. Presentation of the HSPB1 expressions in various clinical subgroups (p > 0.05). Figure 8. Based on the GSE7803 dataset, the ROC analysis and expression validation of the biomarkers. A: ROC. B: The expression of the biomarkers. [file 41065_2025_418_MOESM1_ESM.zip › Supplementary Figures/Supplementary Figure2.tif]

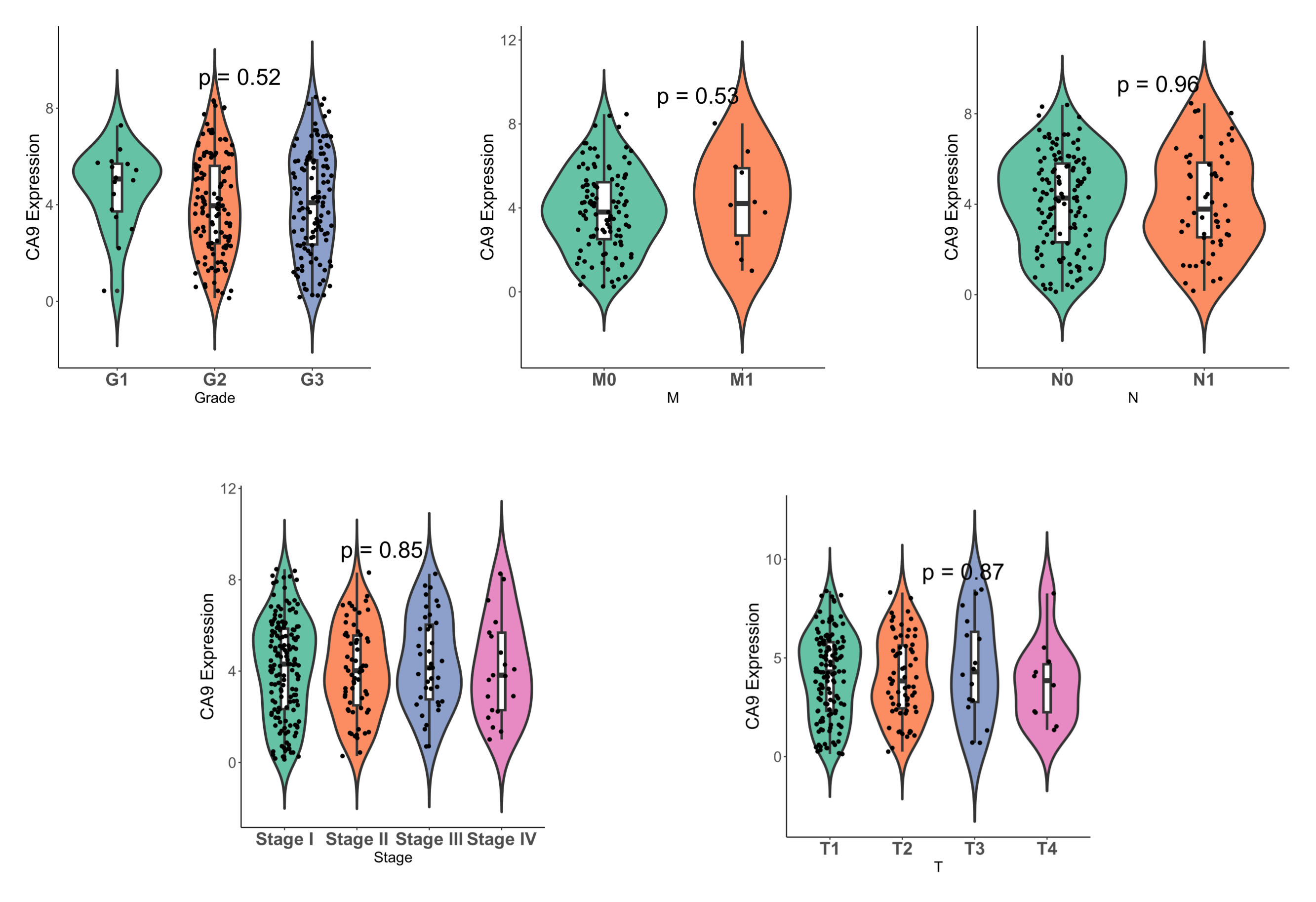

Supplement: Supplementary file 1 — Supplementary Material 1: Figure 1. Sankey diagram of the interaction between N6-methyladenosine (m6A)-related genes and m6A-related ferroptosis genes (MRFGs). Left: m6A; right: MRFGs. Figure 2. Presentation of the ALOX12 expressions in various clinical subgroups (p > 0.05). Figure 3. Presentation of the CA9 expressions in various clinical subgroups (p > 0.05). Figure 4. Presentation of the CDC25A expressions in various clinical subgroups (p > 0.05). Figure 5. Presentation of the CDCA3 expressions in various clinical subgroups (p > 0.05). Figure 6. Presentation of the EZH2 expressions in various clinical subgroups (p > 0.05). Figure 7. Presentation of the HSPB1 expressions in various clinical subgroups (p > 0.05). Figure 8. Based on the GSE7803 dataset, the ROC analysis and expression validation of the biomarkers. A: ROC. B: The expression of the biomarkers. [file 41065_2025_418_MOESM1_ESM.zip › Supplementary Figures/Supplementary Figure3.tif]

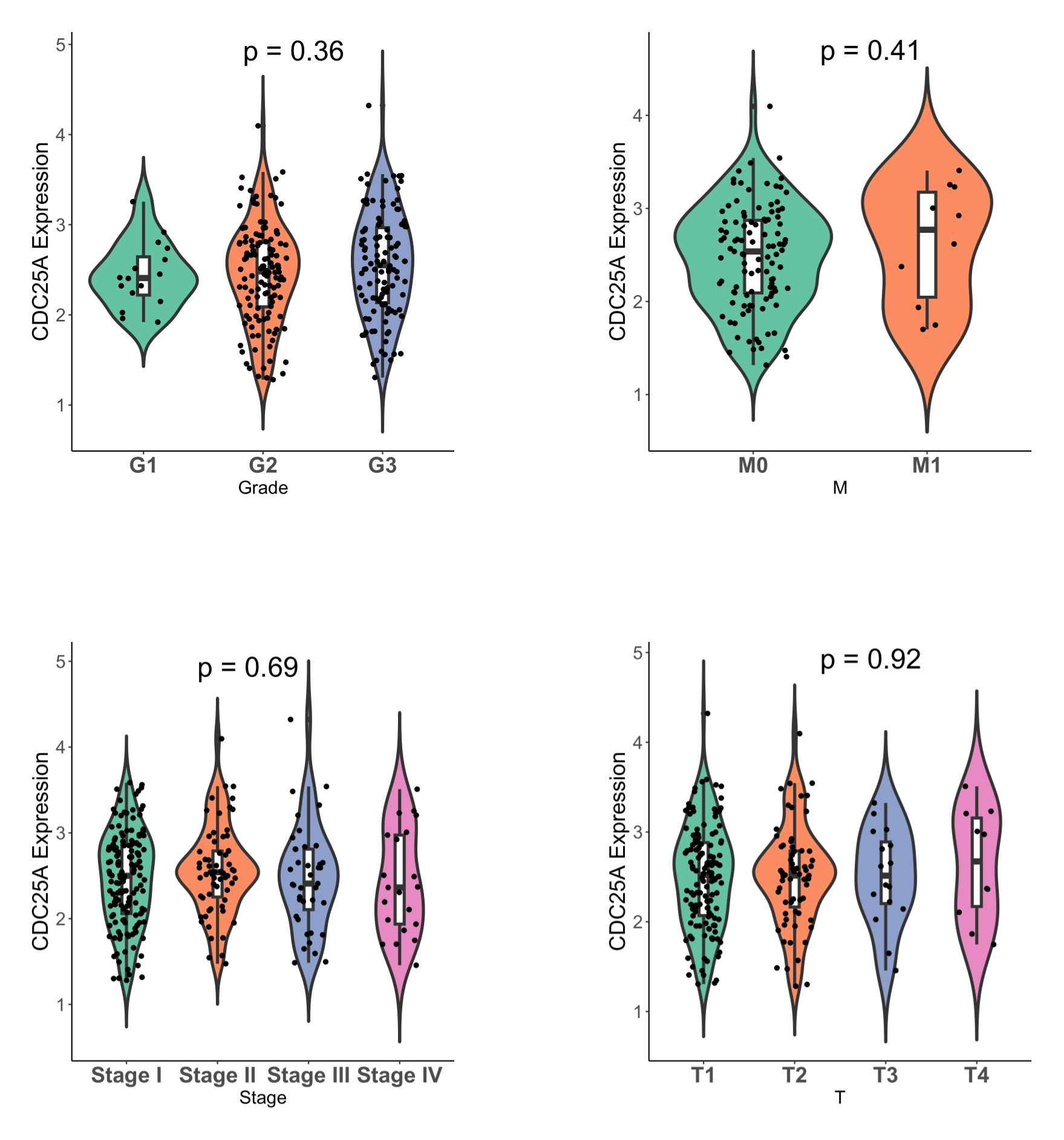

Supplement: Supplementary file 1 — Supplementary Material 1: Figure 1. Sankey diagram of the interaction between N6-methyladenosine (m6A)-related genes and m6A-related ferroptosis genes (MRFGs). Left: m6A; right: MRFGs. Figure 2. Presentation of the ALOX12 expressions in various clinical subgroups (p > 0.05). Figure 3. Presentation of the CA9 expressions in various clinical subgroups (p > 0.05). Figure 4. Presentation of the CDC25A expressions in various clinical subgroups (p > 0.05). Figure 5. Presentation of the CDCA3 expressions in various clinical subgroups (p > 0.05). Figure 6. Presentation of the EZH2 expressions in various clinical subgroups (p > 0.05). Figure 7. Presentation of the HSPB1 expressions in various clinical subgroups (p > 0.05). Figure 8. Based on the GSE7803 dataset, the ROC analysis and expression validation of the biomarkers. A: ROC. B: The expression of the biomarkers. [file 41065_2025_418_MOESM1_ESM.zip › Supplementary Figures/Supplementary Figure4.tif]

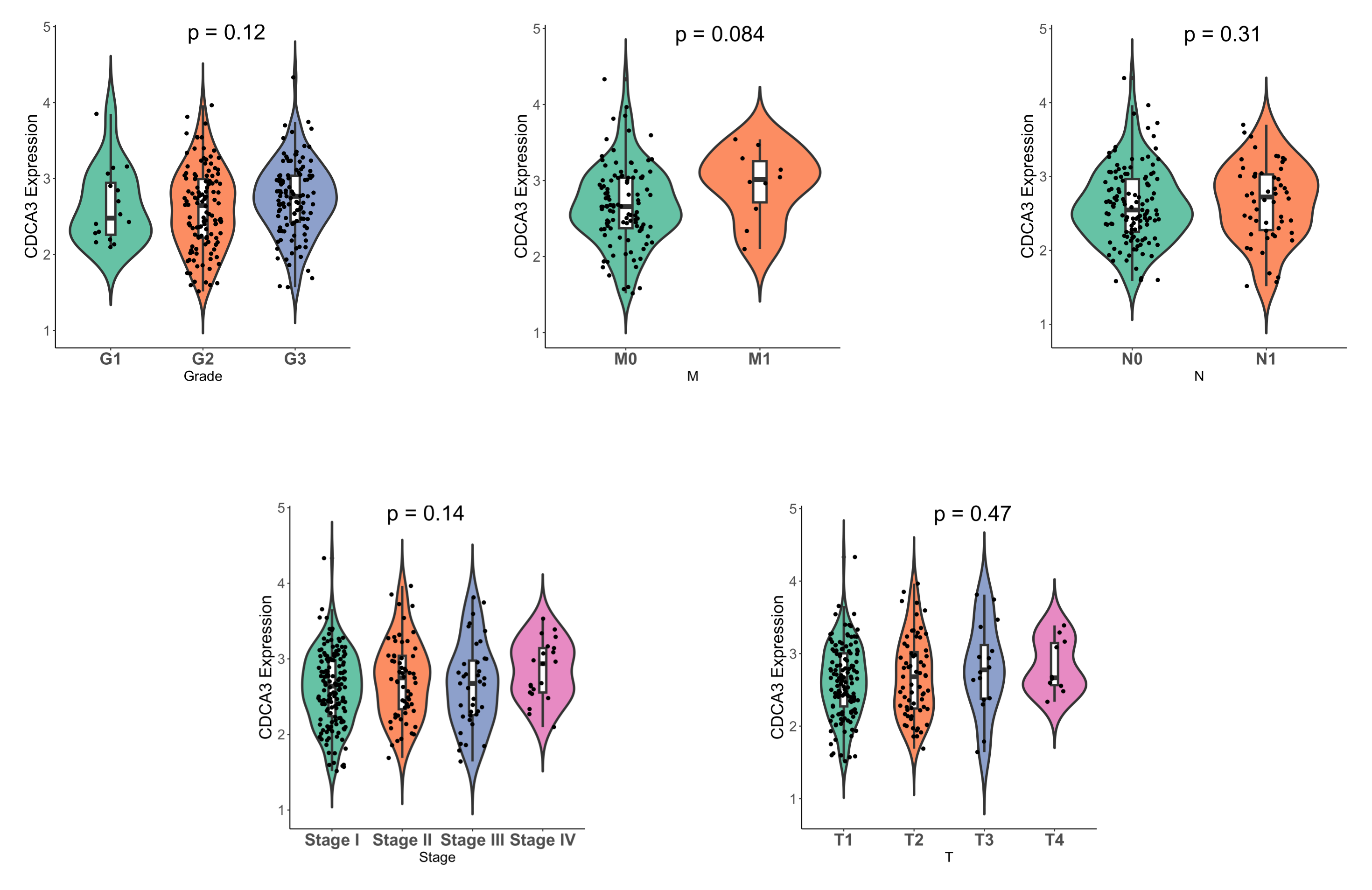

Supplement: Supplementary file 1 — Supplementary Material 1: Figure 1. Sankey diagram of the interaction between N6-methyladenosine (m6A)-related genes and m6A-related ferroptosis genes (MRFGs). Left: m6A; right: MRFGs. Figure 2. Presentation of the ALOX12 expressions in various clinical subgroups (p > 0.05). Figure 3. Presentation of the CA9 expressions in various clinical subgroups (p > 0.05). Figure 4. Presentation of the CDC25A expressions in various clinical subgroups (p > 0.05). Figure 5. Presentation of the CDCA3 expressions in various clinical subgroups (p > 0.05). Figure 6. Presentation of the EZH2 expressions in various clinical subgroups (p > 0.05). Figure 7. Presentation of the HSPB1 expressions in various clinical subgroups (p > 0.05). Figure 8. Based on the GSE7803 dataset, the ROC analysis and expression validation of the biomarkers. A: ROC. B: The expression of the biomarkers. [file 41065_2025_418_MOESM1_ESM.zip › Supplementary Figures/Supplementary Figure5.tif]

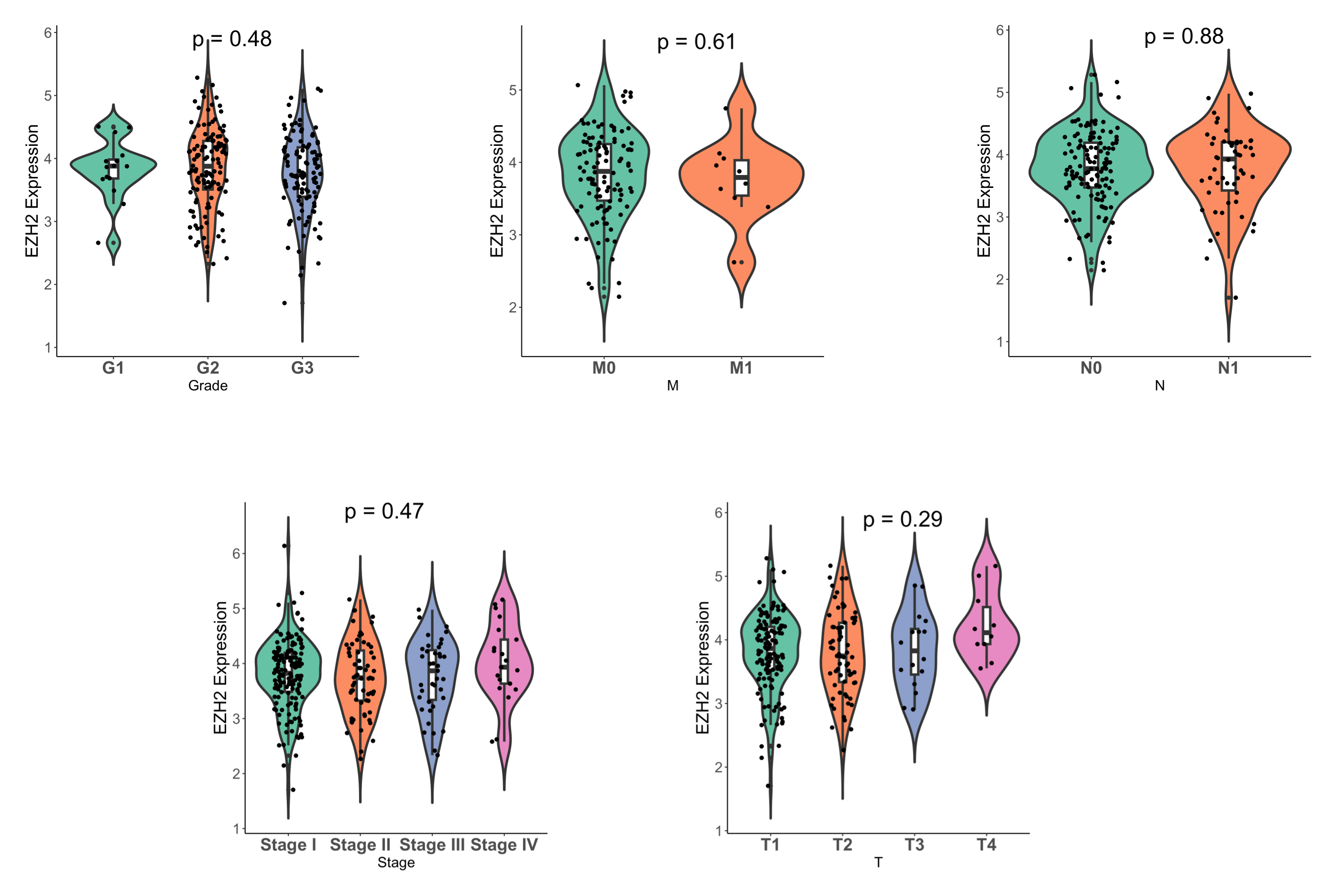

Supplement: Supplementary file 1 — Supplementary Material 1: Figure 1. Sankey diagram of the interaction between N6-methyladenosine (m6A)-related genes and m6A-related ferroptosis genes (MRFGs). Left: m6A; right: MRFGs. Figure 2. Presentation of the ALOX12 expressions in various clinical subgroups (p > 0.05). Figure 3. Presentation of the CA9 expressions in various clinical subgroups (p > 0.05). Figure 4. Presentation of the CDC25A expressions in various clinical subgroups (p > 0.05). Figure 5. Presentation of the CDCA3 expressions in various clinical subgroups (p > 0.05). Figure 6. Presentation of the EZH2 expressions in various clinical subgroups (p > 0.05). Figure 7. Presentation of the HSPB1 expressions in various clinical subgroups (p > 0.05). Figure 8. Based on the GSE7803 dataset, the ROC analysis and expression validation of the biomarkers. A: ROC. B: The expression of the biomarkers. [file 41065_2025_418_MOESM1_ESM.zip › Supplementary Figures/Supplementary Figure6.tif]

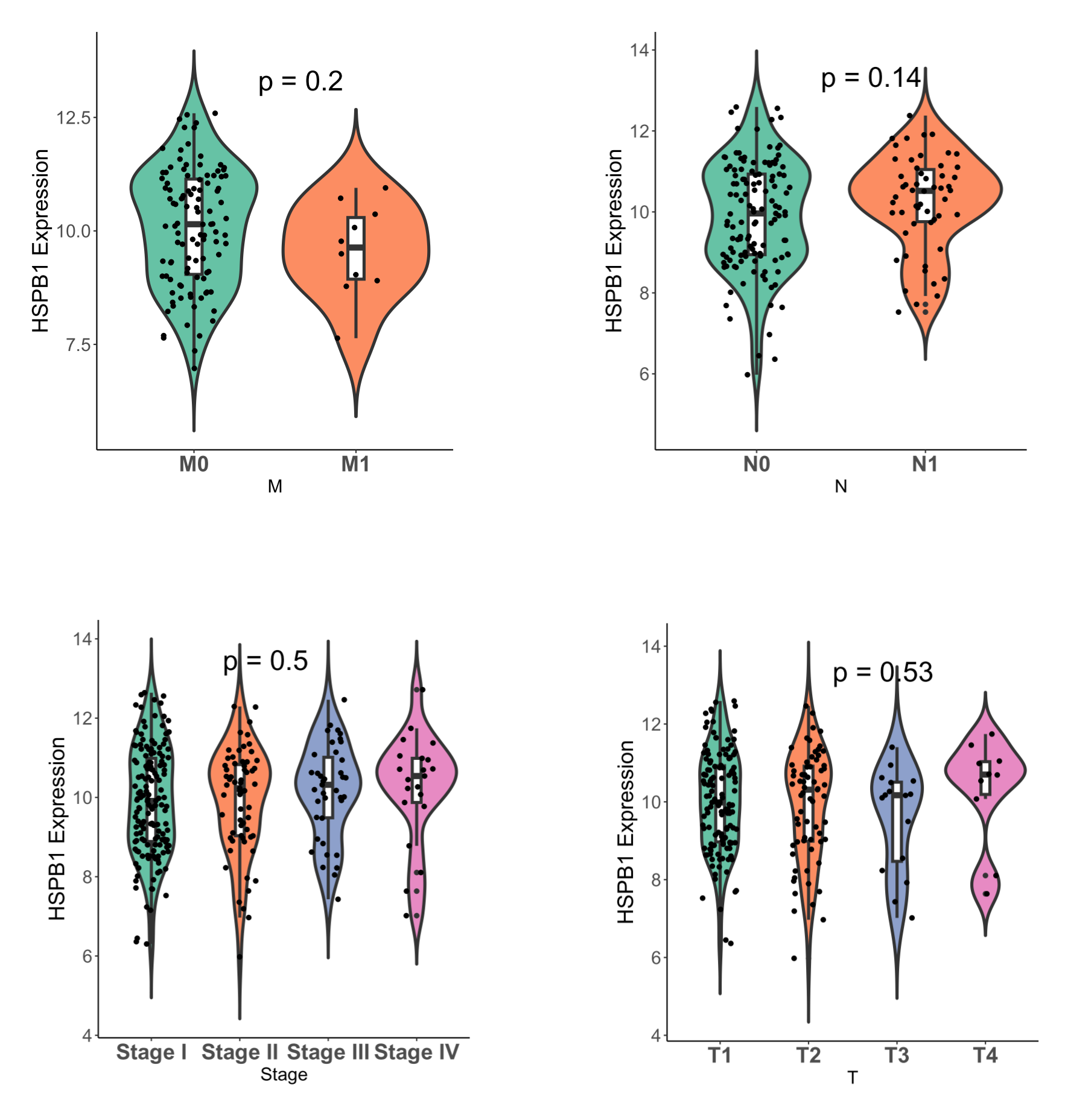

Supplement: Supplementary file 1 — Supplementary Material 1: Figure 1. Sankey diagram of the interaction between N6-methyladenosine (m6A)-related genes and m6A-related ferroptosis genes (MRFGs). Left: m6A; right: MRFGs. Figure 2. Presentation of the ALOX12 expressions in various clinical subgroups (p > 0.05). Figure 3. Presentation of the CA9 expressions in various clinical subgroups (p > 0.05). Figure 4. Presentation of the CDC25A expressions in various clinical subgroups (p > 0.05). Figure 5. Presentation of the CDCA3 expressions in various clinical subgroups (p > 0.05). Figure 6. Presentation of the EZH2 expressions in various clinical subgroups (p > 0.05). Figure 7. Presentation of the HSPB1 expressions in various clinical subgroups (p > 0.05). Figure 8. Based on the GSE7803 dataset, the ROC analysis and expression validation of the biomarkers. A: ROC. B: The expression of the biomarkers. [file 41065_2025_418_MOESM1_ESM.zip › Supplementary Figures/Supplementary Figure7.tif]
